# The conserved ribonuclease aCPSF1 triggers genome-wide transcription termination of Archaea via a 3′-end cleavage mode

**DOI:** 10.1093/nar/gkaa702

**Published:** 2020-08-28

**Authors:** Lei Yue, Jie Li, Bing Zhang, Lei Qi, Zhihua Li, Fangqing Zhao, Lingyan Li, Xiaowei Zheng, Xiuzhu Dong

**Affiliations:** State Key Laboratory of Microbial Resources, Institute of Microbiology, Chinese Academy of Sciences, Beijing 100101, China; University of Chinese Academy of Sciences, No. 19A Yuquan Road, Shijingshan District, Beijing 100049, China; State Key Laboratory of Microbial Resources, Institute of Microbiology, Chinese Academy of Sciences, Beijing 100101, China; University of Chinese Academy of Sciences, No. 19A Yuquan Road, Shijingshan District, Beijing 100049, China; Beijing Institutes of Life Science, Chinese Academy of Sciences, Beijing 100101, China; State Key Laboratory of Microbial Resources, Institute of Microbiology, Chinese Academy of Sciences, Beijing 100101, China; State Key Laboratory of Microbial Resources, Institute of Microbiology, Chinese Academy of Sciences, Beijing 100101, China; University of Chinese Academy of Sciences, No. 19A Yuquan Road, Shijingshan District, Beijing 100049, China; University of Chinese Academy of Sciences, No. 19A Yuquan Road, Shijingshan District, Beijing 100049, China; Beijing Institutes of Life Science, Chinese Academy of Sciences, Beijing 100101, China; State Key Laboratory of Microbial Resources, Institute of Microbiology, Chinese Academy of Sciences, Beijing 100101, China; State Key Laboratory of Microbial Resources, Institute of Microbiology, Chinese Academy of Sciences, Beijing 100101, China; State Key Laboratory of Microbial Resources, Institute of Microbiology, Chinese Academy of Sciences, Beijing 100101, China; University of Chinese Academy of Sciences, No. 19A Yuquan Road, Shijingshan District, Beijing 100049, China

## Abstract

Transcription termination defines accurate transcript 3′-ends and ensures programmed transcriptomes, making it critical to life. However, transcription termination mechanisms remain largely unknown in Archaea. Here, we reported the physiological significance of the newly identified general transcription termination factor of Archaea, the ribonuclease aCPSF1, and elucidated its 3′-end cleavage triggered termination mechanism. The depletion of *Mmp-aCPSF1* in *Methanococcus maripaludis* caused a genome-wide transcription termination defect and disordered transcriptome. Transcript-3′end-sequencing revealed that transcriptions primarily terminate downstream of a uridine-rich motif where *Mmp*-aCPSF1 performed an endoribonucleolytic cleavage, and the endoribonuclease activity was determined to be essential to the *in vivo* transcription termination. Co-immunoprecipitation and chromatin-immunoprecipitation detected interactions of *Mmp*-aCPSF1 with RNA polymerase and chromosome. Phylogenetic analysis revealed that the aCPSF1 orthologs are ubiquitously distributed among the archaeal phyla, and two aCPSF1 orthologs from *Lokiarchaeota* and *Thaumarchaeota* could replace *Mmp*-aCPSF1 to terminate transcription of *M. maripaludis*. Therefore, the aCPSF1 dependent termination mechanism could be widely employed in Archaea, including *Lokiarchaeota* belonging to Asgard Archaea, the postulated archaeal ancestor of Eukaryotes. Strikingly, aCPSF1-dependent archaeal transcription termination reported here exposes a similar 3′-cleavage mode as the eukaryotic RNA polymerase II termination, thus would shed lights on understanding the evolutionary linking between archaeal and eukaryotic termination machineries.

## INTRODUCTION

Transcription, the fundamental biological process for transforming the genetic information from DNA to RNA, must be properly terminated (1–3). The proper terminating transcription is essential to (i) guarantee accurate transcript 3′-ends, (ii) prevent read-through resulted undesired increase of downstream gene or antisense transcriptions, (iii) recycle RNA polymerase (RNAP) and (iv) minimize collision of biomacromolecular machineries (2–4). Consequently, organisms have evolved precise mechanisms to govern transcription termination (2–5). Bacteria primarily employ Rho-dependent and -independent (intrinsic) termination mechanisms, with the former relying on an RNA translocase Rho to dissociate the transcription elongation complex (TEC), while the latter merely depends on a nascent RNA structure harboring a 7–8 base-paired hairpin followed by a run of uridine residues (2,3,6,7). In eukaryotes, RNAP II, which transcribes mRNAs and non-coding RNAs, employs multifaceted termination strategies but usually with an involvement of transcript 3′-end processing event. In this event, the poly(A) site, one of the 3′-end processing/termination signals in nascent RNA, is recognized by a cleavage and polyadenylation complex (CPF/CPSF); thereafter, an endoribonuclease in CPF/CPSF, the human CPSF73 or the yeast Ysh1, cleaves the RNA downstream. This processing event is followed by polyadenylation for mRNA maturation, and triggers RNAP II dissociation for transcription termination (3,5). Additionally, efficient dismantling RNAP II requires other termination factors (3,5,8–12).

However, the transcription termination mechanisms in the third life form, Archaea, remain largely unknown (1,13). Archaea represent a primary domain of cellular life that consists of highly diverse prokaryotic phyla, and phylogenetically are closer to eukaryotes (14–16). Specifically, Archaea employ an eukaryotic RNAP II homolog, the archaeal RNAP (aRNAP) (17), but have compact genomes with short intergenic-regions (IGRs) and usually co-transcribed polycistronic operons. Therefore, transcription in Archaea must be properly terminated to prevent readthrough resulted transcription interference of the downstream gene. Earlier studies on individual genes have revealed that Archaea possess a bacteria-like but distinct intrinsic termination mode, by depending merely on a short uridine-rich sequence but not strictly requiring an upstream hairpin structure (1,18–22). Recently, the genome-level 3′-ends sequencing (Term-seq) analyses on three representative archaeal species, *Methanosarcina mazei*, *Sulfolobus acidocaldarius* and *Haloferax volcanii*, have identified similar over-representative uridine-rich tracts and without preceding hairpin structures in the transcripts 3′-ends (23,24). Term-seq also identified the eukaryotic-like alternative 3′-untranslated regions (3′-UTRs) in Archaea that are generated by consecutive termination sites, implying that Archaea could employ eukaryotic-like transcription termination modes and may also employ trans-acting (termination) factors (13). Eta has been reported to release stalled TECs from damaged DNA sites in *Thermococcus kodakarensis* (25), however it functions specially in response to DNA damages (1,25,26). Very recently, FttA, an archaeal ribonuclease aCPSF1 from *T. kodakarensis*, was reported in mediating transcription termination on a cytidine-rich sequence in an *in vitro* system (27), but the physiological significances of aCPSF1 mediated termination and the working mechanisms remain largely unknown.

In the present work, we report that the aCPSF1 ortholog, *Mmp*-aCPSF1 of *Methanococcus maripaludis*, depending on its ribonuclease activity, triggers a genome-wide transcription termination and ensures programmed transcriptome and optimal growth, so identifying aCPSF1 as a general transcription termination factor of Archaea. A phylogenetic analysis reveals that aCPSF1 is ubiquitously distributed in the archaeal phyla, and two orthologs from distant related *Lokiarchaeota* and *Thaumarchaeota* were experimentally verified to implement transcription termination in *Methanococcus*. Thus, the aCPSF1 dependent transcription termination could be widely employed in Archaea. Noticeably, aCPSF1 mediates the archaeal transcription termination via a transcript 3′-end cleavage mode, resembling its eukaryotic orthologs (yeast Ysh1 and human CPSF73) in eukaryotic RNAP II transcription termination.

## MATERIALS AND METHODS

### Strains, plasmids and culture conditions

Strains and plasmids used in this study are listed in Supplementary Table S1. *M. maripaludis* S2 and its derivatives were grown in pre-reduced McF medium under a gas phase of N_2_/CO_2_ (80:20) at 37 and 22°C as previously described (28), and 1.5% agar was used in the solid medium. Puromycin (2.5 μg/ml), neomycin (1.0 mg/ml) and 8-azahypoxanthine (0.25 mg/ml) were used for genetic modification selections unless indicated otherwise. *Escherichia coli* DH5α, BL21(DE3)pLysS and BW25113 were grown at 37°C in Luria-Bertani (LB) broth and supplemented with ampicillin (100 μg/ml) or streptomycin (50 μg/ml) when needed.

### Construction of the *Mmp-aCPSF1* gene depletion and complementary strains

Given the possible essentiality of *Mmp-aCPSF1* (*MMP0694*) determined in *M. maripaludis* (29) and repeated failures in deletion of *Mmp-aCPSF1*, the *Mmp-aCPSF1* expression depleted strain (▽*aCPSF1*) was constructed using a *TetR*-*tetO* repressor-operator system as follows.

First, a tetracycline responsive regulator (*tetR*) and operator (*tetO*) cassette from *Bacillus subtilis* was fused upstream to the N-terminal His_6_-tagged *Mmp*-*aCPSF1*, and integrated at the *hpt* locus of *M. maripaludis* S2 to construct a tetracycline regulated *Mmp*-*aCPSF1* overexpression strain *tetO-aCPSF1* by 8-azahypoxanthine sensitivity selection. To achieve gene expression in *M. maripaludis*, the promoter (P*mcr*) and terminator (T*mcr*) of the methyl-CoM reductase were respectively fused to the *tetR* gene at the 5′- and 3′-termini and inserted to pMD19-T (TAKARA) to obtain pMD19-T-P*mcr*-*tetR*-T*mcr* (Supplementary Table S1). The *tetO* operator was positioned downstream the nitrogen fixation gene *nif* promoter (P*nif*) and fused upstream the amplified *Mmp-aCPSF1* ORF to obtain a *tetO* fused *Mmp-aCPSF1* fragment P*nif*-*tetO*-His_6_-*aCPSF1*. For homologous recombination at the *hpt* site, two DNA fragments, each of approximately 800-bp respectively from the upstream and downstream *hpt*, was amplified from the S2 genomic DNA and fused with the P*mcr*-*tetR*-T*mcr* and P*nif*-*tetO*-His_6_-*aCPSF1* fragment at each end to obtain the plasmid of p*tetR*/*tetO*-His_6_-*aCPSF1* (Supplementary Table S1) via the stepwise Gibson assembly using ClonExpress MultiS One Step Cloning Kit (Vazyme). The PCR fragment amplified from the plasmid p*tetR*/*tetO*-His_6_-*aCPSF1* using primers *Hpt*up-F/*Hpt*down-R (Supplementary Table S2) was transformed into S2 using the polyethylene glycol (PEG)-mediated transformation approach (30) to generate a tetracycline regulated *Mmp*-*aCPSF1* overexpression strain (*tetO-aCPSF1*) via double cross homologous recombination. Transformants were selected by plating on McF agar containing 8-azahypoxanthine as described previously (31), and confirmed via PCR amplification using primers *Hpt*up-F/*Hpt*down-R.

Next, an in-frame deletion of the indigenous *Mmp*-*aCPSF1* gene was implemented in strain *tetO-aCPSF1*. Two ∼800-bp DNA fragments, each from the upstream and downstream the *Mmp*-*aCPSF1* gene was PCR amplified and fused to the puromycin-resistance cassette *pac* gene amplified from the plasmid pIJA03 at each end to generate the plasmid pMD19T-Δ*aCPSF1* via the stepwise Gibson assembly. The DNA fragment of *aCPSF1*up-*pac*-*aCPSF1*down amplified from pMD19T-Δ*aCPSF1* was transformed into strain *tetO-aCPSF1* to knockout the indigenous *Mmp*-*aCPSF1*. Transformants were selected on McF agar containing puromycin, and the *Mmp-aCPSF1* gene deletion was validated by PCR amplification. Thus, a tetracycline regulated *Mmp-aCPSF1* expression strain ▽*aCPSF1* was obtained.

To obtain various ▽*aCPSF1* complementary strains, the respective *aCPSF1* orthologs or mutated gene were cloned into the *Xho*I/*Bgl* II sites of the replicative plasmid pMEV2 to produce pMEV2-*aCPSF1*(wt)/pMEV2-*aCPSF1*(mu) carrying the wild-type *Mmp-aCPSF1* and its mutant, H243A/H246A, respectively, and pMEV2-*Lokiarch44440*/pMEV2*-CENSYa1545*, carrying two codon optimized *Mmp-aCPSF1* orthologs from Ca. *Lokiarchaeum* sp. GC14_75 and Ca. *Cenarchaeum symbiosum*. The constructed plasmids were each transformed into ▽*aCPSF1* via the PEG-mediated transformation approach to produce the complementary strains.

### Construction of the in-frame deletion mutants of *earA* in strains S2 and ▽*aCPSF1*

The in-frame deletion mutants of *earA* were constructed similarly as above. Two DNA fragments, each of ∼800-bp respectively from the upstream and downstream *earA* (*MMP1718*), were amplified and fused to a neomycin-resistance cassette amplified from the plasmid pMEV2 at each end. The resulting *earA*up-*neo*-*earA*down fragment was transformed into strains S2 and ▽*aCPSF1*, and via double cross homologous recombination, the *earA* deletion mutants Δ*earA* and ▽*aCPSF1*Δ*earA* (Supplementary Table S1) were obtained, respectively.

### Construction of Flag-tagged *Mmp-*aCPSF1 and His-Flag-tagged *Mmp-*RpoL fusion strains

3 × Flag or His_6_ and 3 × Flag tags were firstly fused to the C termini of *Mmp*-aCPSF1 and *Mmp*-RpoL via PCR amplification and cloned into pMD19-T to construct the plasmids pMD19-T-*aCPSF1*–3Flag and pMD19-T-*rpoL*-3Flag-His_6_, respectively. The puromycin or neomycin cassette in the plasmid pIJA03 or pMEV2 was amplified and overlapping fused to the 800-bp fragment downstream of *Mmp-aCPSF1* or *Mmp-RpoL*. The overlapped PCR fragments were then inserted into downstream of *Mmp*-*aCPSF1*–3Flag or *Mmp*-*rpoL*-3Flag-His_6_ to obtain pMD19-T-*aCPSF1*–3Flag-*pac* and pMD19-T-*rpoL*-3Flag-His_6_-*neo* (Supplementary Table S1), respectively. Next, DNA fragments were amplified from pMD19-T-*aCPSF1*–3Flag-*pac* and pMD19-T-*rpoL*-3Flag-His_6_-*neo* using primers *aCPSF1*Flag-F/*aCPSF1*Flagdw-R and *RpoL*Flag-F/*RpoL*Flagdw-R, respectively, and transformed into *M. maripaludis* S2 to obtain Flag-tagged strains of *Mmp*-aCPSF1-F and *Mmp*-RpoL-HF, respectively.

### Extraction of genomic DNA and total RNA

The genomic DNA of *M. maripaludis* S2 and its derivatives was extracted and purified from the mid-exponential 37°C-grown cultures (OD_600_ = 0.5) using TIANamp Bacteria DNA Kit (TIANGEN Biotech, Beijing, China) by following the protocol instruction. Total RNA used for RNA-seq, Term-seq, northern blotting, and rapid amplification of cDNA 3′-ends was extracted from the mid-exponential cultures of 22°C-grown strains S2, *Mmp*-com(*Mmp*-C1), *Mmp*-com(*Loki*-C1) and *Mmp*-com(*Csy*-C1) (OD_600_ = 0.45), strains ▽*aCPSF1* and *Mmp*-com(*Mmp*-C1mu)) (OD_600_ = 0.36) and 37°C-grown strains S2 and ▽*aCPSF1* (OD_600_ = 0.5) using TRIzolTM reagent (Invitrogen). Quantification and qualification of the purified RNA were determined using the NanoPhotometer spectrophotometer (IMPLEN, CA, USA), Qubit 2.0 Flurometer (Life Technologies, CA, USA) and Agilent Bioanalyzer 2100 system (Agilent Technologies, CA, USA).

### RNA-seq and data analysis

Using the methods described previously (32), high-throughput strand-specific RNA-seq was performed for differential transcriptome analysis between strains S2 and ▽aCPSF1 with three biological replicates. The library quality of the RNA samples was assessed on an Agilent Bioanalyzer 2100 system. Library construction and Illumina sequencing was performed at Novogene Bioinformatics Technology Co., Ltd (Beijing, China). rRNA was depleted using Ribo-Zero™ rRNA Removal Kit (Epicentre). The raw RNA-seq reads were first subjected to adapter sequence trimming and then QC preprocessing to discard those meeting any of the following thresholds: truncated reads of ≤1/3 length of the original ones, reads containing ≥10% uncalled bases (Ns), or ≥50% low-quality bases (PHRED quality scores ≤ 5). Next, the QC filtered reads were mapped to the reference genome of *M. maripaludis* S2 (GCF_000011585) using Bowtie2 (v2.2.3) (-x refer.fa -1 1.clean.fq -2 2.clean.fq –fr –no-unal -S mapped.sam) (33) after removing the reads aligned to the reference rRNAs (Supplementary Table S3). HTSeq (v0.6.1) (-m union -s reverse -f bam -t exon) (34) was used to count the read numbers mapped to each gene. Differential expression analysis between strain S2 and ▽*aCPSF1* was performed on un-normalized read counts using the DESeq2 (35) (v1.20.0) algorithm, and differentially expressed genes were defined for those having the Padj, an adjusted *P*-value, <0.05. The FPKM of each gene was calculated based on the fragment numbers per kilobase of transcript sequence per millions base pairs sequenced that mapped to the gene (36).

To calculate the genome-wide transcriptional read-through index (TRTindex), transcriptional units (TUs) were first predicted using Rockhopper (v2.0.3) (37) on the transcriptomic data of the wild-type strain S2. Intergenic regions (IGRs) were defined as the DNA region between the transcription termination site (TTS) of a TU to the transcription start site (TSS) of the tandem downstream TU on the same strand. Transcript abundances (FPKMs) of each TU and IGR were determined by HISAT2 (v2.0.5) (38) using parameters (–no-spliced –rna-strandness RF) and quantified using StringTie (v1.3.3) (39) with –e option, and the predicted TU and IGR regions were transformed to the GTF file format used as –G parameter in StringTie (40). TRTindex was calculated using the four criteria shown in Figure 2e and listed in Supplementary Dataset S1.

### Term-seq and data analysis

Term-seq libraries were constructed using the procedures described previously (23). Sequencing was performed on an Illumina HiSeq X-ten system with 150-bp paired-end reads. Raw Term-seq data were filtered using the similar QC steps as described above. Then, QC filtered reads (Supplementary Table S5) were aligned to the reference genome of *M. maripaludis* S2 (GCF_000011585) using Bowtie2 (33). The read counts of the 3′-ends mapped to each genomic position in strain S2 were recorded. The primary TTSs were assigned based on four criteria: (i) within 200 nt downstream the stop codon of a gene; (ii) >1.1 read ratio of -1 site (predicted TTS) to +1 site (1 nt downstream TTS); (iii) read-counts of −1 site minus +1 site > 5; (iv) upon (ii) and (iii) satisfied, the site that had the highest read count difference of −1 site minus +1 site and also occurred in the two biological replicates (Supplementary Dataset S2). Using WebLogo(v2.8.2) (41), the terminator logos were created on the sequences (Supplementary Dataset S2) flanking Term-seq predicted TTS. To compare the read differences flanking the primary TTSs in strains S2 and ▽*aCPSF1*, a metaplot analysis was performed on the average reads of each 20 nt upstream and downstream of all the primary TTSs, which were normalized by dividing the individual average read of each site from −21 to +20 by that of -21 site shown in Figure 4A.

### Rapid amplification of cDNA 3′-ends (3′RACE)

3′RACE was performed as described previously (42). A total of 20 μg of total RNA were ligated with 50 pmol 3′RACE Linker (5′-rAppCTGTAGGCACCATCAAT–NH_2_–3′; NEB) through a 16 h incubation at 16°C with 20 U T4 RNA ligase (Ambion). The 3′ linker-ligated RNA was recovered by isopropanol precipitation and one aliquot of 2 μg was mixed with 100 pmol 3′R-RT-P (5′-ATTGATGGTGCCTACAG-3′, complementary to the 3′RACE linker) by incubation at 65°C for 10 min and on ice for 2 min. Then, it was used in the reverse transcription (RT) reaction using 200 U SuperScript III reverse transcriptase (Invitrogen). After RT, nested PCR was conducted using primers (Supplementary Table S2) that target regions of <200 nt upstream the termination codons to obtain gene-specific products. Specific PCR products were excised from 2% agarose gel and cloned into pMD19-T (TaKaRa) and then sequenced. The 3′-end of a TU is defined as the nucleotide linked to the 3′RACE-linker.

### Protein purification

For purification of the *Mmp*-aCPSF1 protein, a His-tagged SUMO-*aCPSF1* fusion was first constructed and cloned into pSB1s using the ClonExpress MultiS One Step Cloning Kit. Plasmid pSB1s that carries His-SUMO-*aCPSF1* was then transformed into *E. coli* BW25113 and the transformants were induced by 0.1% l-arabinose. After a 16-h induction at 22°C, the cells were harvested, and the protein was purified as described previously (43). Next, His-SUMO tags were removed by incubating with His-tagged Ulp1 protease at 30°C for 2 h, and the *Mmp*-aCPSF1 protein was purified through a His-Trap HP column to remove His-tagged SUMO and His-tagged Ulp1. The Rpo D/L protein of *M. maripaludis* S2 (*Mmp*-aRpoD/L) was overexpressed and purified as described previously (44). In brief, the ORFs of *Mmp*-*rpoD* (*MMP1322*) and *Mmp*-*rpoL* (*MMP0262*) were stepwise cloned into pGEX-4T-1 to obtain the plasmid pGEX-4T-1-*rpoD/L*, and transformed into *E. coli* BL21(DE3)pLysS for overexpression. Purified proteins were detected by SDS-PAGE, and the protein concentration was determined using a BCA protein assay kit (Thermo Scientific).

### Nuclease activity assay

Four RNA fragments containing the termination sites and the flanking sequences of *MMP1100, MMP1149*, *MMP0901* and *MMP1697* were synthesized by Genscript (China). They were 5′-end labeled with [γ-^32^P] ATP (PerkinElmer) using T4 polynucleotide kinase (Thermo Scientific). A 10 μl nuclease reaction mixture contained 20 μM wild-type *Mmp*-aCPSF1 protein or its catalytic inactive mutant, 5 nM 5′-[γ-^32^P] labeled RNA, 20 mM HEPES, (pH 7.5), 150 mM NaCl, 5 mM MgCl_2_ and 5% (w/v) glycerol. Nucleolytic reactions were initiated by the addition of the protein at 37°C for 90 min and stopped by 15 min incubation with 10 μg/ml Proteinase K (Ambion) at 55°C. The reaction products were then mixed with formamide-containing dye and analyzed on 10% sequencing urea-PAGE. A nucleotide ladder was generated by alkaline hydrolysis of the labeled RNA substrates. The urea-PAGE gels were analyzed by autoradiography with X-ray film.

### Western blot assay

Western blot was performed to determine the cellular *Mmp*-aCPSF1 or *Mmp*-Rpo D/L protein abundances in various genetic modified strains. A polyclonal rabbit antiserum against the purified *Mmp*-aCPSF1 or *Mmp*-Rpo D/L protein was raised by MBL International Corporation, respectively. The mid-exponential cells of *M. maripaludis* were harvested and resuspended in a lysis buffer [50 mM Tris–HCl (pH 7.5), 150 mM NaCl, 10 (w/v) glycerol, 0.05% (v/v) NP-40], and lysed by sonication. The cell lysate was centrifuged and proteins in the supernatant were separated on 12% SDS-PAGE and then transferred to a nitrocellulose membrane. The antisera of anti-*Mmp*-aCPSF1 (1: 8000) and anti-*Mmp*-aRpoD/L (1: 20 000) were diluted and used respectively, and a horseradish peroxidase (HRP)-linked secondary conjugate at 1:5000 dilutions was used for immunoreaction with the anti-*Mmp*-aCPSF1 or anti-*Mmp*-aRpoD/L antiserum. Immune-active bands were visualized by an Amersham ECL Prime Western blot detection reagent (GE Healthcare). Protein quantification was performed using Quantity One (Bio-Rad).

### Northern blot and mRNA half-life analysis

Northern blot was performed to determine various cellular RNA species and contents as described previously (45). To measure the half-life of a specific transcript, a final concentration of 100 μg/ml actinomycin D (actD, MP Biomedicals) was added into the exponential cultures of *M. maripaludis* to stop transcription. At 0, 5, 10, and 15 min post-addition of actD, each 3 ml culture was collected and the RNA was isolated. The RNA content in each sample was quantified by northern blot using the corresponding labeled DNA probe. A linear regression plot based on the average RNA contents from three batches of culture plotted against the sampling time was used for calculation of the half-life of decay (*t*_1/2_) that was the time point when 50% RNA content was retained.

To determine the bulk mRNA half-life, *M. maripaludis* was cultured to the exponential phase, and 30 μCi [5,6–^3^H]-Uridine (PerkinElmer) was added to isotope label the nascent transcripts. After a 10 min-labeling, actD was added and each 600 μl of the culture were collected at 0, 2, 4, 6, 8 and 10 min post-addition of actD as described above. Then, 67 μl of 100% cold trichloroacetic acid (TCA) was added to precipitate the total RNA, and the mixture was incubated on ice for >30 min. A sample of 60 min post-addition of act D was used for counting the stable RNAs. RNA in each TCA mixture was precipitated by centrifuge at 12 000 g for 10 min and dissolved in 700 μl of Ecoscint A (National Diagnostics) for ^3^H isotope counting using liquid scintillator detector (PerkinElmer). The stable RNA ^3^H counts were first deducted from the total ^3^H ones at each sampling time point and the resultant count at 0 min post-addition of act D was recorded as 100% mRNA, and the residual mRNA content at each time point was plotted against the sampling time. The bulk mRNA half-life was calculated as described above.

### Electronic microscopy and motility assay

The mid-exponential cells of *M. maripaludis* strains S2 and ▽*aCPSF1* grown at 22°C or 37°C were loaded onto carbon-Formvar-coated copper grids and ddH_2_O washed, and stained with uranyl acetate. Cells and archaella were observed under an LKB-V Ultratome (Jel1400) transmission electronic microscope. For motility assay, cells were collected from a 5-ml stationary phase culture grown at 22°C or 37°C, and then resuspended gently in 200 μl of McF medium. 10 μl of cell suspension were inoculated in the semi-solid McF medium containing 0.25% (w/v) agar inside an anaerobic chamber. Plates were incubated at 22°C for 15 or 20 days or 37°C for 2 days in an anaerobic jar with Oxoid AnaeroGen (Thermo Scientific) to remove the oxygen.

### Size exclusion chromatography

The exponential cells of *M. maripaludis* S2 were harvested and resuspended in the lysis buffer (50 mM Tris–HCl (pH 7.5), 150 mM NaCl, 0.05% NP-40 and 10% (v/v) glycerol), and then lysed by sonication and centrifuged at 12 000g for 30 min at 4°C. The supernatant was incubated at 37°C for 30 min with and without treatment of DNase I (100 U), RNase A (100 U) or Benzonase Nuclease (100 U). A total of 500 μg of the pretreated supernatant was fractionated through the Superdex 200 10/300 GL column (GE Healthcare) and the molecular sizes of each fraction was estimated by gel filtration molecular weight markers (Kit No.: MWGF1000 of Sigma-Aldrich). Finally, each fraction was collected for protein identification by western blot.

### Co-immunoprecipitation and tandem affinity chromatography (TAP)

The association of *Mmp*-aCPSF1 with *Mmp*-aRNAP was detected by co-immunoprecipitation and tandem affinity chromatography. To perform the anti-Flag co-immunoprecipitation, prewashed anti-FLAG M2 magnetic beads (Sigma) was added to 5 mg of pretreated cell extract and incubated 8 h at 4°C with gently shaking. The antigen bound magnetic beads were washed five times with the lysis buffer and eluted by 2.5 volumes of 3× FLAG peptide (150 ng/μl, Sigma) for 3 h at 4°C with gently shaking. For the His-tag affinity chromatography, about 20 mg of cell extract were purified through a His-Trap HP column as described above. For TAP, about 20 mg of cell extract were first purified by His-tagged affinity chromatography, and the product was concentrated and imidazole removed using Amicon Ultrafra-30 concentrators (Millipore). The concentrated product was then immunoprecipitated using the same procedure as for anti-Flag co-immunoprecipitation. All of the CoIP-, His-tagged affinity chromatography- and TAP-products were separated on a 12% SDS-PAGE gel and identified by Western blot.

### Chromatin Immunoprecipitation (ChIP) coupled quantitative PCR (ChIP-qPCR)

To obtain the chromosomal occupancy of *Mmp*-aCPSF1 and *Mmp-aRpoL*, ChIP assays were performed for strains *Mmp*-aCPSF1-F and *Mmp*-aRpoL-HF as described previously (46–48) with some modifications. Cells of *M. maripaludis* S2 were used as mock controls. About 0.5–1 × 10^9^ middle exponential cells ml^−1^ were formaldehyde fixed and lysed. The chromosomal DNA in the lysed supernatant was sonicated to an average size of 200–500 bp using Bioruptor UCD300 (Diagenode). After overnight incubation with anti-FLAG M2 Magnetic Beads (Sigma) at 4°C, the beads were washed and the DNA-protein complexes were eluted by the ChIP elution buffer (10 mM Tris–HCl (pH 8.0), 1 mM EDTA, 1% SDS) at 65°C for 30 min with shaking. Reverse crosslinking was then performed by a treatment of 0.05 mg/ml RNase A and then 0.5 mg/ml proteinase K at 37°C for 2–4 h and followed by overnight incubation at 65°C. DNA fragments were purified using MiniElute columns (Qiagen) and quantified using the Qubit dsDNA HS kit (Life Technologies).

PCR and qPCR were performed to determine the DNA species and the enrichment by *Mmp*-aCPSF1 or *Mmp*-aRpoL as described previously (47,49). For PCR amplification, each 1 μl of input and IP or mock-IP DNA sample was used in a 25 μl reaction mix containing 400 nM of the corresponding oligonucleotide primer (Supplementary Table S2). For qPCR analysis, a similar reaction mixture of PCR except for 1× SYBR Green Real-time PCR Master Mix (Toyobo, Tokyo, Japan) was used and performed at Mastercycler EP realplex2 (Eppendorf, Germany). Enrichment folds between *Mmp*-aCPSF1 or *Mmp*-aRpoL and mork-IP samples were calculated as: ΔCt value (normalized to the input samples) for each sample was calculated according to the equation ΔCt = [Ct (sample) – adjusted Ct (input)]. Then, ΔΔCt was calculated through the equation ΔΔCt = (ΔCt (experimental sample) – ΔCt (mock sample)). Finally, the enrichment fold between experimental sample and mock sample was calculated using 2^(–ΔΔCt)^. ChIP assays were performed for three biological triplicates, and PCR and qPCR were assayed for technical triplicates of each biological repeat.

### Uridine-rich tracts searching

For searching uridine-rich tracts in the selected representative archaeal genomes (Supplementary Dataset S3), we downloaded the reference genomes and annotation files from the NCBI database (https://www.ncbi.nlm.nih.gov/search/). Samtools (version1.9) (50) faidx option was used to retrieve the sequence in length of 200 nt upstream and downstream the stop codons of the putative ORFs in the archaeal genomes, and then the U-rich tracts with successive five, or six or seven uridine bases (U5, U6 and U7) within the sequence were counted using an in-house script.

## RESULTS

### Depleted expression of *Mmp*-aCPSF1 causes growth reduction and a disordered transcriptome

**Figure 1.**
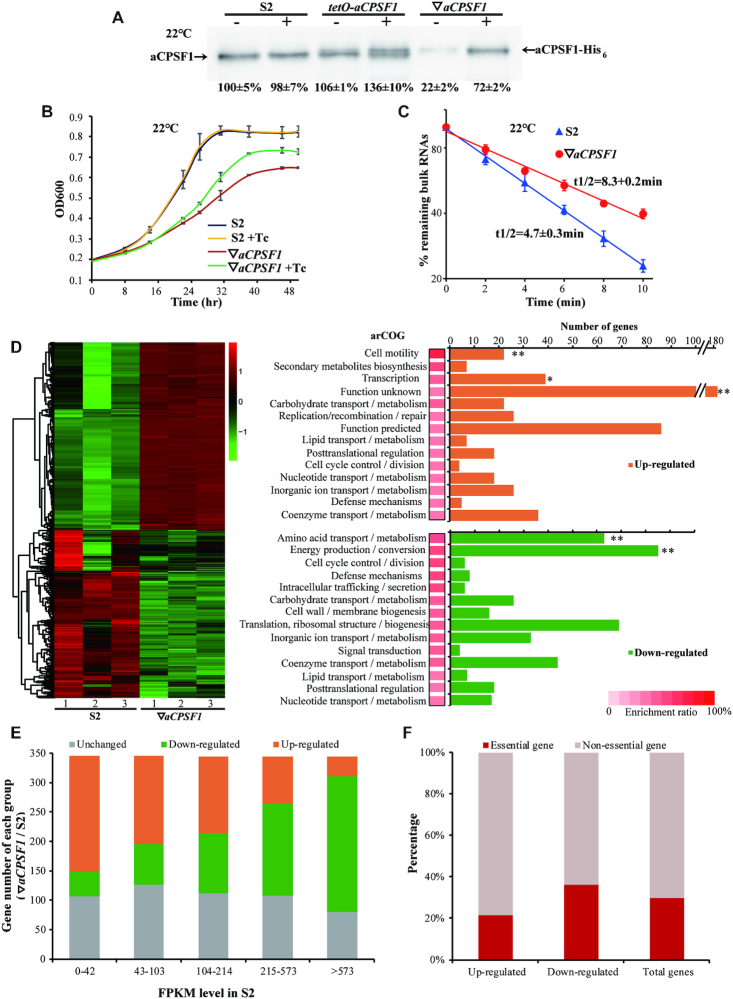
Depleted expression of *Mmp-aCPSF1* results in reduced growth, prolonged RNA lifespan, and a disordered transcriptome of 22°C-cultured *M. maripaludis*. (**A**) Western blot assayed *Mmp*-aCPSF1 protein abundance (percentages referenced to lane 1) with (+) or without (–) 100 μg/ml of tetracycline in 22°C-grown S2 (wild-type), *tetO-aCPSF1*, and ▽*aCPSF1*. Averages and standard deviations of three replicates are shown. aCPSF1 and aCPSF1-His_6_ flanking the gel point the indigenous- and *hpt*-site inserted His_6_-*Mmp*-aCPSF1, respectively. The schematic depicting the construction of *tetO-aCPSF1* and ▽*aCPSF1* was shown in Supplementary Figure S1A. (**B, C**) Depletion of *Mmp*-*aCPSF1* reduced 22°C-growth (**B**) and prolonged the bulk mRNA half-lives (**C**). Three batches of cultures of strains S2 and ▽*aCPSF1* with or without tetracycline (Tc) were measured, and the averages and standard deviations are shown. Bulk mRNA half-lives were determined by quantifying the [^3^H]-uridine signal attenuation. (**D**) Hierarchical clustering (left) and functional category (right) analysis of the differential transcribed genes in three batches of 22°C-cultures of S2 and ▽*aCPSF1*. Heat plot representation of the differential expression ratio (log_2_) is shown with color intensity. Green and red represent the minima and maxima fold, respectively. The functional category enrichment ratio (a bar with gradient red intensity at lower right corner) was percentages of the up (orange)- and down (green)-regulated gene numbers (horizontal axis) in total genes of each arCOG categories (Supplementary Table S4). The significance statistical analysis was analyzed by Fisher's exact test with Benjamini-Hochberg multiple-testing correction to calculate P-values for each function category, and ** and * respectively mark the significant enriched categories with *P* < 0.01 and < 0.05 (Supplementary Table S4). (**E**) Hierarchical grouping of the differential transcribed genes caused by *Mmp-aCPSF1* depletion. Based on the FPKMs in strain S2, transcripts were grouped into five ranks (<42, 43–103, 104–214, 215–573 and >573 of FPKMs), and then the up- and down-regulated and unchanged transcript numbers in each rank in ▽*aCPSF1* were counted. (**F**) According to the gene essentiality in *M. maripaludis* defined by Sarmiento *et al.* (29), percentages of essential and non-essential genes falling in the up- and down-regulated and unchanged groups in ▽*aCPSF1* were calculated, respectively.

The archaeal CPSF1 affiliates with the β-CASP family of ribonucleases, and the endoribonuclease activities were identified in three aCPSF1 orthologs (51–53), with one also exhibiting 5′–3′ exoribonuclease activity (52) via the *in vitro* assays. The aCPSF1 orthologs are universally distributed in all detected archaeal phyla (52), and *Mmp*-*aCPSF1* was determined to be an essential gene in *M. maripaludis* by Sarmiento *et al.* (29) and by our repeated failures in deletion of the gene as well, highlighting the fundamental roles of aCPSF1 in Archaea. To investigate the physiological functions of aCPSF1, the *Mmp*-aCPSF1 expression depleted strain (▽*aCPSF1*) was constructed in *M. maripaludis* by using the *TetR*-*tetO* repressor-operator system (Supplementary Figure S1A). Compared with the wild-type strain S2, only 60% and 20% of the *Mmp*-aCPSF1 protein abundances were determined in the 37°C- and 22°C-grown ▽*aCPSF1* (minus tetracycline), respectively, verifying a successful depletion of *Mmp-aCPSF1* (Supplementary Figure S1B and Figure 1A). Although having a similar growth rate as the wild-type strain S2 at 37°C (Supplementary Figure S1C), ▽*aCPSF1* exhibited markedly reduced growth at 22°C (*μ* = 0.035 h^−1^ versus 0.06 h^−1^ of S2, Figure 1B), corresponding to the three-fold lower protein abundance of *Mmp*-aCPSF1 in 22°C- than in 37°C-grown ▽*aCPSF1*. Concomitantly, an ∼2-fold longer bulk mRNA half-life was determined in the 22°C-cultured ▽*aCPSF1* than that of S2 (8.4 min versus 4.7 min, Figure 1C), suggesting that aCPSF1 could have an effect on archaeal mRNA turnover.

Differential transcriptomic analysis based on strand-specific RNA-seq analysis revealed that the depletion of *Mmp*-*aCPSF1* had changed transcriptions of about 69% genes (1188) in 22°C-grown cultures with each half up- and down-regulated (Figure 1D and Supplementary Dataset S4). Among the up-regulated genes, those belonging to the arCOGs of cell motility and transcription were significantly enriched, while the down-regulated genes fell mainly into the arCOGs of energy production and amino acid metabolism, conforming to the reduced growth of ▽*aCPSF1*. By hierarchically grouping all transcripts into five ranks based on their FPKMs in strain S2, we found that the transcript abundances of the highly and lowly expressed ranks were reversely changed in ▽*aCPSF1*, namely that the highly expressed genes were generally down-regulated, while the poorly expressed ones were mostly up-regulated (Figure 1E), behaving like ‘robbing the rich to feed the poor’ caused by *Mmp-aCPSF1* depletion. In addition, about 40% of Sarmiento *et al.* (29) defined essential genes decreased expression and only 20% increased expression in ▽*aCPSF1* (Figure 1F). This indicates that *Mmp*-aCPSF1 plays a role in the control of the ordered transcriptome.

### *Mmp-aCPSF1* depletion results in a genome-wide transcription read-through

**Figure 2.**
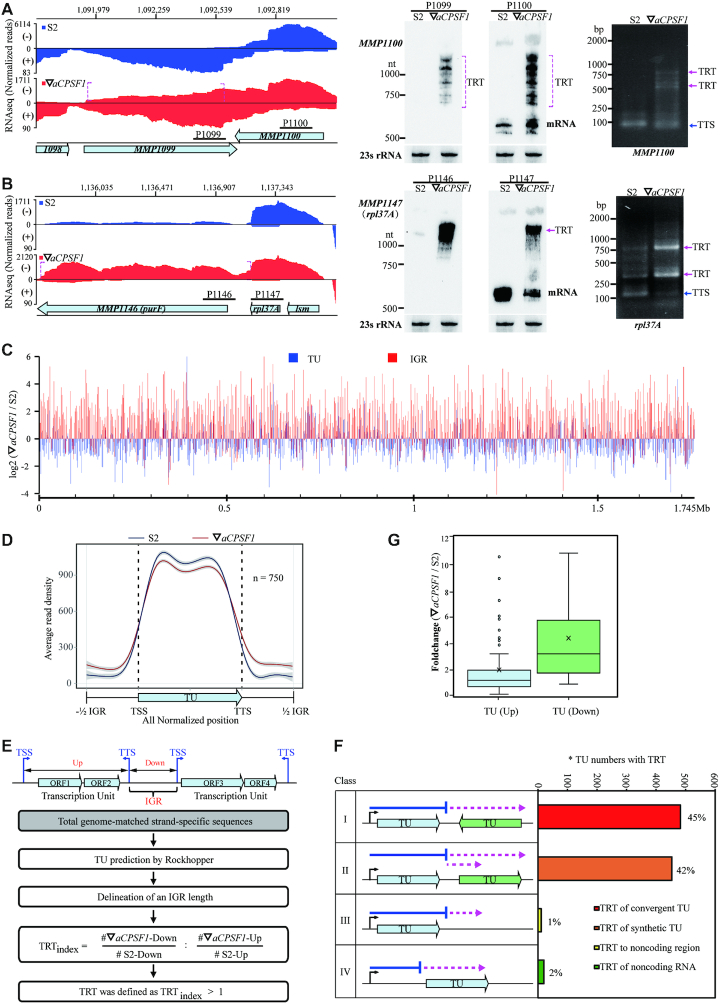
The depletion of *Mmp-aCPSF1* causes a genome-wide transcription read-through (TRT). (**A, B**) The strand-specific RNA-seq mapping profiles (left) show 3′-end extensions (dot magenta brackets) of *MMP1100* (**A**) and *MMP1147* (**B**) in ▽*aCPSF1* (red) referenced to that in strain S2 (blue). Numbers on top indicate the genomic sites, and bullets show genes. Northern blot (middle) assayed TRTs using the respective probes (horizontal sticks in A), and 23S rRNA was used as an internal control. 3′RACE (right) assayed transcription termination sites (TTSs) and TRTs. (**C**) Pair-wise comparison of genome-wide Log2 FPKM ratios of transcription units (TUs) and the intergenic regions (IGRs) between strain S2 and ▽*aCPSF1*. The beneath ruler indicates the genomic location. (**D**) A metaplot diagram shows the average reads mapping pattern of TUs and the flanking IGRs in S2 and ▽*aCPSF1* based on normalization of 750 TUs that have an IGR length >100 nt. (**E**) A flowchart depicts TRT identification and TRT_index_ calculation. (**F**) A diagram shows percentages of each TRT type occurred in ▽*aCPSF1*. Bent arrows and horizontal blue lines indicate TSS and transcript lengths in strain S2, respectively, and dot magenta arrows indicate transcripts that occur TRT in ▽*aCPSF1*. *, TU numbers occurring TRTs. (**G**) Boxplots show the FPKM fold changes of Type II TRT in (F) for the upstream TUs that generate TRTs (TU (Up)) and the tandem downstream TUs (TU (Down)), respectively.

Strikingly, by querying the strand-specific RNA-seq mapping files, we observed pervasive prolonged transcript 3′-ends, i.e. transcription read-throughs (TRTs) in ▽*aCPSF1*. Such as *MMP1100*, a predicated master regulator for methanogenesis (54), its 3′-end was markedly extended and multiple-reads mapped, and so resulted in an antisense transcription of *MMP1099* on the opposite strand (Figure 2A, left); the 3′-end of the upstream *MMP1147* was extended into the downstream *MMP1146* on the same strand as well (Figure 2B, left). Northern blot (Figure 2A, B and Supplementary Figure S2, middle) and rapid amplification of the cDNA 3′-ends (3′RACE) (Figure 2 A, B and Supplementary Figure S2 right) verified the TRTs in ▽*aCPSF1*. The *M. maripaludis* genome comprises 1722 coding genes that are organized into 1079 predicted transcription units (TUs) with a median 200 bp of IGRs (Supplementary Dataset S1). A global pair-wise comparison was performed for the FPKMs of a TU and its IGR in S2 and ▽*aCPSF1* and a metaplot diagram was visualized through normalizing those of 750 TUs, which displayed a genome-wide FPKM increase in IGRs but reduction in TU bodies upon *aCPSF1* depletion (Figure 2C, D and Supplementary Dataset S1). Further statistical analysis on 1079 TUs in ▽*aCPSF1* and S2 yielded higher folds of elevated transcription in IGRs (median: 4.36-fold) than in TU bodies (median: 1.21-fold) (Supplementary Figure S3). Thus, the *Mmp*-*aCPSF1* depletion has caused a genome-wide TRT.

### TRTs pervasively elevate expressions of the co-directional downstream genes and result in exuberant archaella along with enhanced motility of *M. maripaludis*

**Figure 3.**
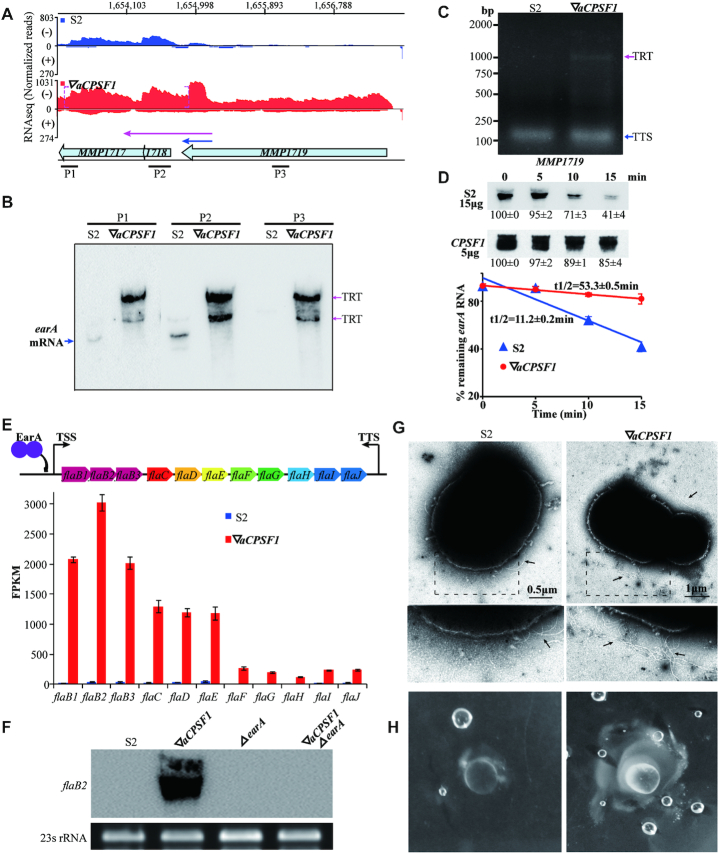
TRT upregulates the archaella regulator *earA* and results in exuberant archaella along with elevated motility in 22°C-cultured ▽*aCPSF1*. (**A**) A diagram shows the RNA-seq reads mapped to *earA* (*MMP1718*) and the adjacent genes in S2 (blue) and ▽*aCPSF1* (red). Dot magenta brackets show that *Mmp-aCPSF1* depletion caused 3′-end extension. Numbers on top indicate the mapped genomic regions, and bullets represent the corresponding genes. (**B**) Northern blot assays *MMP1719* TRT into *earA* and *MMP1717* by using DNA probes P1, P2 and P3 shown in (A). (**C**) 3′RACE amplified fragments contain the transcription termination site (TTS) in S2 and TRT of *MMP1719* in ▽*aCPSF1*, respectively. (**D**) Northern blot assays the *earA* transcript stability in S2 and ▽*aCPSF1* (upper panel) using probe P2. Hybridization signal intensities were estimated using Quantity One (v4.6.6) and the residual percentages at indicated times referenced to that at 0 min were shown at beneath. Half-lives (t1/2) were calculated from the regression curve of remnant mRNA contents along time (lower panel). Experiments were performed on three batches of cultures, and the averages and standard deviations are shown. (**E**) A schematic depicts gene organization of the *fla* operon and its activator EarA (upper). TTS is located 104 nt downstream the stop codon (nt 1618850) of *fla J*. The histograms (lower) show the FPKMs of the *fla* operon genes in S2 and ▽*aCPSF1*. (**F**) Northern blot assays the *flaB2* transcript abundances in strains S2, ▽*aCPSF1*, *earA* deletion (Δ*earA*), and the double mutation of *Mmp*-*aCPSF1* depletion and *earA* deletion (▽*aCPSF1*Δ*earA*). 23S rRNA was used as the sample control. (**G**) Representative electron microscopic images show more archaella (arrows pointed) at ▽*aCPSF1* than S2 cells (upper panels) and in the magnified regions (dot framed, lower panels). Archaella were observed for each 20 cells, and additional representative images are shown in Supplementary Figure S5. (**H**) Mobility assay displays larger lawn and more bubbles in ▽*aCPSF1* than that in S2 when grown on 0.25% agar.

Next, a pipeline (Figure 2E) was developed to define the *Mmp*-*aCPSF1* depletion caused TRTs via four criteria: (i) prediction of TUs based on the transcriptomic data of strain S2; (ii) delineation of an IGR from the upstream transcription termination site (TTS) to the downstream transcription start site (TSS) of TUs on the same DNA strand; (iii) calculation of TRT_index_ using the following equation,

$$\begin{eqnarray*} {\boldsymbol{TRTindex}} &=& \frac{{\# \triangledown aCPSF1 - {\rm{Down}}}}{{\# {\rm{S}}2 - {\rm{Down}}}} \nonumber \\ && :\frac{{\# \triangledown aCPSF1 - {\rm{Up}}}}{{\# {\rm{S}}2 - {\rm{Up}}}} \end{eqnarray*}$$

where *#*▽*aCPSF1-* and #S2-Down indicate the IGR FPKMs in ▽*aCPSF1* and S2, respectively; #▽*aCPSF1*- and #S2-Up refer to the TU FPKMs in ▽*aCPSF1* and S2, respectively; 4) defining a TRT by its TRT_index_ >1. Based on the criteria, TRT was identified in 965 TUs (89.5% of 1079 TUs) in ▽*aCPSF1*, of which 706 TUs (65.7% of 1079 TUs) had a TRT_index_ >2. According to TRTs’ locations and directions, four TRT types were classified, i.e. TRT of an upstream TU extending into the oppositely encoded one and producing antisense transcripts (type I, 45%), or into the downstream co-directional TU (type II, 42%), or into the downstream IGR (type III, 1%); and TRT of an IGR noncoding RNA extending into the downstream TU (type IV, 2%) (Figure 2F). Although type I TRTs did not markedly affect the abundances of convergently encoded TUs on genome-wide (Supplementary Figure S4), type II TRTs noticeably increased the transcriptions (median: 3.4-fold) of the tandem downstream TUs (Supplementary Dataset S1) compared to that of their own (median: 1.3-fold) in ▽*aCPSF1* (Figure 2G), so can severely disturb downstream gene expression and likely bring physiological changes. This was exemplified as that the *MMP1719* TRT increased transcription of the downstream archaella master regulator, *earA* (*MMP1718*) (Figure 3A and Supplementary Dataset S4).

Using three probes that are respectively complementary to *MMP1717*, *earA* and *MMP1719*, northern blot detected a long transcript encompassing *MMP1717*-*earA*-*MMP1719* in 22°C-cultured ▽*aCPSF1*, but only a co-transcribed TU of *MMP1717*-*earA* in S2 (Figure 3B). 3′RACE also verified the *MMP1719* TRT into *earA* and *MMP1717* (Figure 3C). Moreover, a longer mRNA half-life (53.3 min) was determined for the long transcript of *MMP1717*-*earA*-*MMP1719* in ▽*aCPSF1* than that of the short *MMP1717*-*earA* (11.2 min) in S2 (Figure 3D). Thereby, the markedly increased abundance of the *earA* transcript in ▽*aCPSF1* was likely an overlay effect of *MMP1719* TRT upregulated transcription and the elevated stability of the extended transcript. Consistently, the EarA regulated *fla* operon genes (55) were markedly upregulated in ▽*aCPSF1* (Figure 3E and Supplementary Dataset S4), and the increased transcription of *flaB2* disappeared in the double mutation of *Mmp-aCPSF1* depletion and *earA* deletion but occurred in ▽*aCPSF1* (Figure 3F), verifying that TRT resulted upregulation of *earA* increased the *fla* operon transcript abundances in ▽*aCPSF1*. Accordingly, exuberant archaella and more active motility were observed in the ▽*aCPSF1* cells compared with S2 (Figure 3G, H and Supplementary Figure S5). Moreover, 3′RACE also verified the *MMP1719* TRT into *earA* and Northern blot also detected the increased transcription of *flaB2* at 37°C cultured ▽*aCPSF1* stain, and consequently the exuberant archaella and more active motility were also detected in ▽*aCPSF1* cells than S2 at 37°C (Supplementary Figure S6). This determines that the *Mmp-*aCPSF1 dependent transcription termination assures, in addition to optimal growth and ordered transcriptome (Figure 1), the flagellation and motility of the methanoarchaeon.

### Term-seq reveals a uridine-rich terminator motif and verifies a genome-wide TRT caused by *Mmp-aCPSF1* depletion

**Figure 4.**
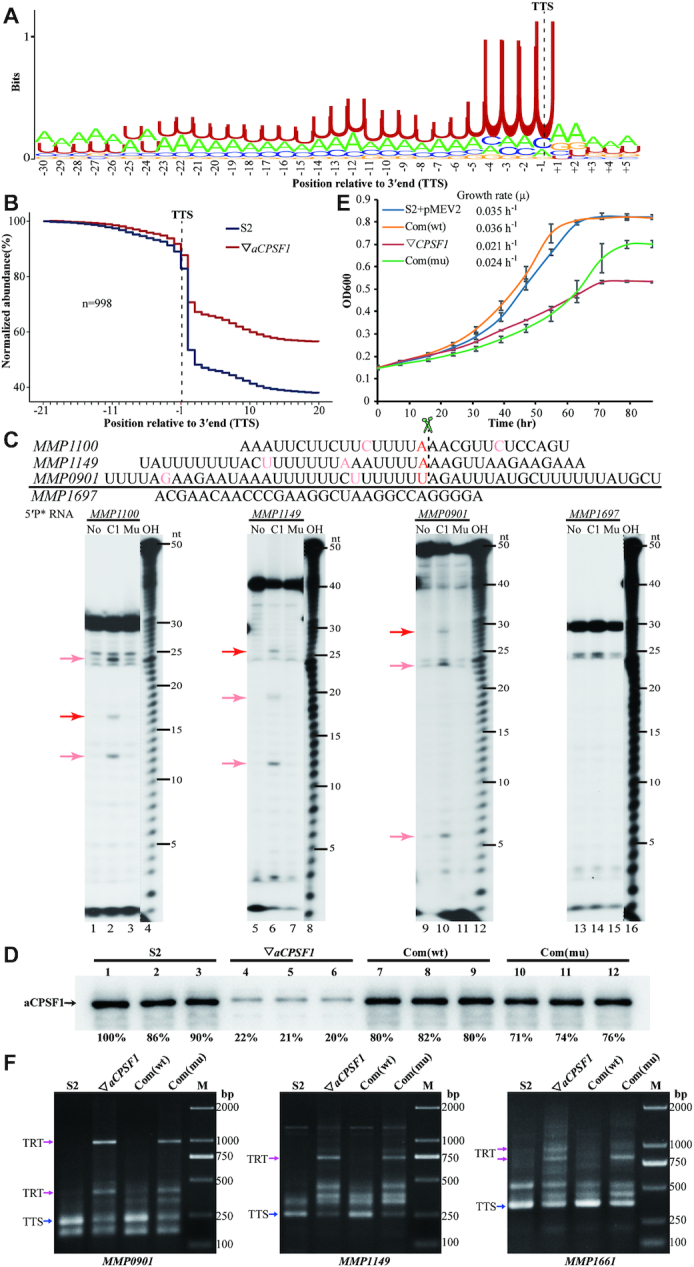
The terminator motif of *M. maripaludis* determined by Term-seq, and the ribonuclease activity of *Mmp*-aCPSF1 on the motif is essential to transcription termination. (**A**) Using WebLogo (v2.8.2) (41), a logo representation was generated for the sequence motif upstream of 998 primary TTSs that are defined by Term-seq. (**B**) A metaplot diagram shows the average reads of each 20 nt upstream and downstream of the 998 primary TTSs (dot black line) in 22°C-cultured S2 (blue line) and ▽*aCPSF1* (red line). (**C**) The ribonuclease activity of *Mmp*-aCPSF1 was assayed on three representative uridine-rich RNAs derived from the TTSs (red bases) flanking sequences of *MMP1100*, *MMP1149* and *MMP0901* (upper). A uridine tract-less RNA fragment from *MMP1697* was used as a control. A urea sequencing gel displays the enzymatic products (lower). No, C1, and Mu indicate the assays without, and with addition of *Mmp*-aCPSF1 and the catalytic inactive mutant (H243A/H246A), respectively. Red and pink arrows point to the presumed cleavage products at TTS and other sites downstream uridine-rich sequences. OH, a hydroxyl ladder indicates migrations of RNA products. (**D**) Western blot assays the *Mmp*-aCPSF1 protein abundances in three batches of 22°C-grown strains S2, ▽*aCPSF1*, *Mmp*-com(*Mmp*-C1) (Com(wt)) and *Mmp*-com(*Mmp*-C1mu) (Com(mu)). (**E**) Growth curves of the four strains were assayed on three batches of the 22°C-grown cultures, and the averages and standard deviations are shown. (**F**) 3′RACE assays the TRTs in 22°C-grown strains S2, ▽*aCPSF1*, Com(wt) and Com(mu). Blue and magenta arrows indicate the PCR products of normal terminations (TTSs) and TRTs, respectively. M, a DNA ladder indicates migrations of the PCR products.

Next, Term-seq was employed to sequence the transcript 3′-ends and determine the terminator characteristics of *M. maripaludis*, as well as the function of *Mmp*-aCPSF1 in transcription termination. Based on a stringent filtration workflow for TTS definition as described in the Materials and Methods, 998 primary TTSs were identified for 960 TUs and 38 non-coding RNAs (Supplementary Dataset S2) representing 67.7% (730) of the predicted 1,079 and newly identified 230 TUs in S2. Analyzing the sequences flanking TTSs revealed a distinct upstream terminator motif, which is characterized by a 23 nt uridine-tract with the TTS most proximal 4 nt having the highest uridine match (Figure 4A and Supplementary Dataset S2). Reads abundance normalization on each 20 nt upstream and downstream of the 998 primary TTSs determined a >50% reads decrease between bases -2 and +2 flanking a TTS in S2, so defined as transcription termination. However, in ▽*aCPSF1*, only <20% of reads abundance decrease between –2 and +2, and markedly higher abundant reads were mapped at the 20 nts downstream TTS (Figure 4B), verifying that aCPSF1 depletion caused a genome-wide TRT at a single-base resolution, i.e. a genome-wide termination defect at the uridine-rich tract. Next, 3′RACE amplification and sequencing of the 3′-extensions in 19 TUs in ▽*aCPSF1* (Figure 2A and Supplementary Figures S2 and S7) validated Term-seq-defined TTSs and the upstream uridine-rich tracts in a single-base accuracy (Supplementary Figure S8). Similar but slighter remarkable TRTs and transcription abundance changes were also detected in selected TU pairs in 37°C-cultured than 22°C-cultured ▽*aCPSF1* strains by northern blot and 3′RACE assays (Supplementary Figure S9). These results collectively demonstrated that the archaeal ribonuclease, aCPSF1, is involved in genome-wide transcription termination.

### *Mmp*-aCPSF1 depends on its ribonuclease activity to trigger transcription termination

Based on the nuclease nature of *Mmp*-aCPSF1, its nucleolytic activity on Term-seq defined terminator motif was then assayed using three representative uridine-rich RNA substrates (5′-[^32^P]-labeled) that are derived from the 3′-ends of *MMP1100* (30 nt), *MMP1149* (40 nt) and *MMP0901* (50 nt) (Figure 4C, upper). Addition of the recombinant *Mmp*-aCPSF1 protein, but not the activity-inactive mutant H243A/H246A (Supplementary Figure S10), produced the cleavage fragments of ∼17 nt, ∼26 nt and ∼29 nt, matching the predicted sizes of products cleaved at downstream the uridine-tract motif in the three synthetic RNAs. However, no cleaving products were found from the non-uridine-tract containing *MMP1697* RNA (Figure 4C). This confirmed the endoribonucleolytic activity of *Mmp*-aCPSF1 at the uridine-tract downstream TTSs. Additional products cleaved downstream other uridine-tracts were also observed (Figure 4C). Nevertheless, *Mmp*-aCPSF1 only exhibited weak *in vitro* ribonuclease activity, implying that interactions with other proteins possibly promote its *in vivo* activity.

To evaluate the contribution of the aCPSF1 ribonuclease activity to transcription termination *in vivo*, the wild-type *Mmp*-*aCPSF1* and its catalytic-inactive mutant, H243A/H246A, were expressed in ▽*aCPSF1* to construct two complementary strains, Com(wt): *Mmp*-com(*Mmp*-C1) and Com(mu): *Mmp*-com(*Mmp*-C1mu) (Supplementary Table S1) respectively. Although comparable cellular *Mmp*-aCPSF1’s abundances were detected (Figure 4D), the Com(mu) was incapable of restoring the 22°C-growth defect of ▽*aCPSF1*, but the Com(wt) can (Figure 4E). Accordingly, 3′RACE determined similar TRTs from *MMP0901*, *MMP1149*, and *MMP1661* in Com(mu) as in ▽*aCPSF1*, but no TRTs were found in Com(wt) (Figure 4F). This collectively determined that *Mmp*-aCPSF1, depending on its ribonuclease activity, triggers transcription termination.

### *Mmp*-aCPSF1 exhibits the *in vivo* association with archaeal RNA polymerase and a chromosomal occupancy at the transcript 3′-ends

**Figure 5.**
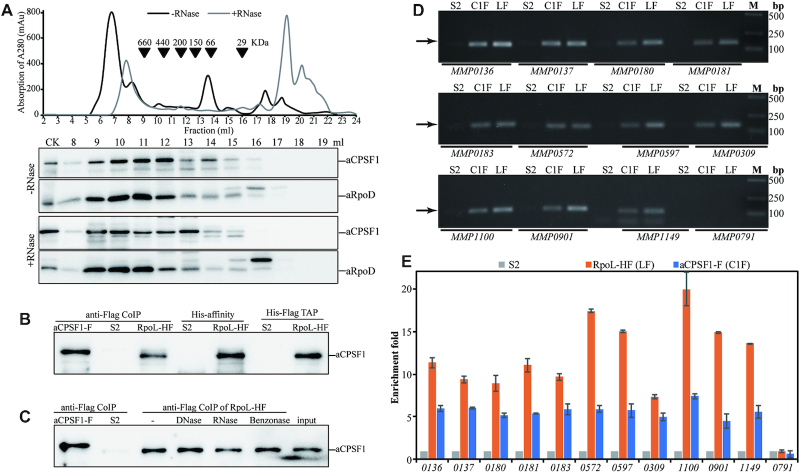
*Mmp*-aCPSF1 associates with *Mmp*-aRNAP and the chromosomal DNA. (**A**) Co-occurrence of *Mmp*-aCFSF1 and *Mmp*-RpoD was assayed through size exclusion chromatography (upper) based cell extract fractionation and coupled with western blot (lower). +/–RNase, with or without RNase A treatment. (**B, C**) Association of *Mmp*-aCPSF1 with *Mmp*-aRNAP was determined through co-immunoprecipitation (anti-Flag CoIP), Ni column affinity chromatography (His-affinity), and tandem affinity purification (TAP) using strains *Mmp*-RpoL-HF and *Mmp*-aCPSF1-F that carry His_6_–3× Flag fused *Mmp*-RpoL and 3× Flag fused *Mmp*-aCPSF1, respectively. Strain S2 was included as a mock control. Western blot detected *Mmp*-aCPSF1 in captured products. -, RNase, DNase, and Benzonase indicate treatments without and with the corresponding nucleases of the *Mmp*-RpoL-HF cell extract before immunoprecipitation. (**D**) PCR products (arrows pointed) of the indicated genes were amplified from the ChIP DNAs of strains S2 (mock), *Mmp*-RpoL-HF (LF), and *Mmp*-aCPSF1-F (C1F), respectively. M, DNA marker. (**E**) qPCR assayed the enriched genes in ChIP products, and the enrichment fold of a given gene in the ChIP eluates of strains *Mmp*-RpoL-HF and *Mmp*-aCPSF1-F over that in S2 (mock control) was calculated as described in the Materials and Methods.

A transcription termination factor would associate with RNAPs *in vivo*. We first investigated the association of *Mmp*-aCPSF1 with *Mmp*-aRNAP by means of size exclusion chromatography-based cell-extract fractionation and coupled with western blot. Co-occurrence of *Mmp*-aCPSF1 and *Mmp*-RpoD, one of the 13 aRNAP subunits of *M. maripaludis*, was found in a protein complex of ∼200–660 kD. Although ribonuclease digestion resulted in some increase of free *Mmp*-aCPSF1 (∼140 kD), the majority of the protein retained in the aRNAP co-occurred complex fractions (Figure 5A), indicating a nucleic-acid independent association of *Mmp*-aCPSF1 with *Mmp*-aRNAP. Furthermore, *Mmp*-aCPSF1 was immunoprecipitated from strain *Mmp*-aCPSF1-F (Supplementary Table S1) that carries the 3× Flag fused *Mmp*-aCPSF1, and it was also detected in the His-affinity purification, co-immunoprecipitated, and tandem affinity chromatography products of strain *Mmp*-RpoL-HF that carries the His_6_–3× Flag fused *Mmp*-RpoL (Figure 5B). RNase, DNase, and Benzonase (digesting both DNA and RNA) treatments did not alter the co-immunoprecipitation results (Figure 5C), further verifying the *in vivo* association of *Mmp*-aCPSF1 and *Mmp*-aRNAP. Moreover, by quantitative western blot, a comparative cellular content of aCPSF1 and RNA polymerase was determined, with aCPSF1 and aRpoL being 0.125 ± 0.009 pmol and 0.072 ± 0.008 pmol at mid-exponential phase and 0.14 ± 0.017 pmol and 0.086 ± 0.006 pmol at stationary phase per μg total cell proteins in 37°C cultures, respectively (Supplementary Figure S11).

Next, the chromosomal DNA occupancy of *Mmp*-aCPSF1 was detected via chromatin immunoprecipitation (ChIP). The chromatin DNAs captured by Flag-tagged *Mmp*-aCPSF1 or aRNAP were immunoprecipitated by ChIP (Supplementary Figure S12) and used as PCR templates. Abundant PCR products in length of 130–150 bp were amplified for 11 highly expressed TUs (Figure 5D), and quantitative PCR (qPCR) determined ∼6- and 6- to 12-fold enrichments of the tested TU 3′-end regions in Flag-tagged *Mmp*-aCPSF1- and *Mmp*-RpoL-captured DNAs, respectively, comparing to that from the mock ChIP of strain S2 (Figure 5E). No PCR products were amplified from the poorly expressed *MMP0791* (Figure 5D, E), and the TTS downstream regions of *MMP0137*, *MMP0180*, *MMP0183* and *MMP1100* (Supplementary Figure S12C). Collectively, *Mmp*-aCPSF1 associates with aRNAP as well as chromosomal DNA *in vivo*, the key features of a transcription termination factor.

### aCPSF1 and the uridine-rich motif within IGRs are widely distributed among Archaea

**Figure 6.**
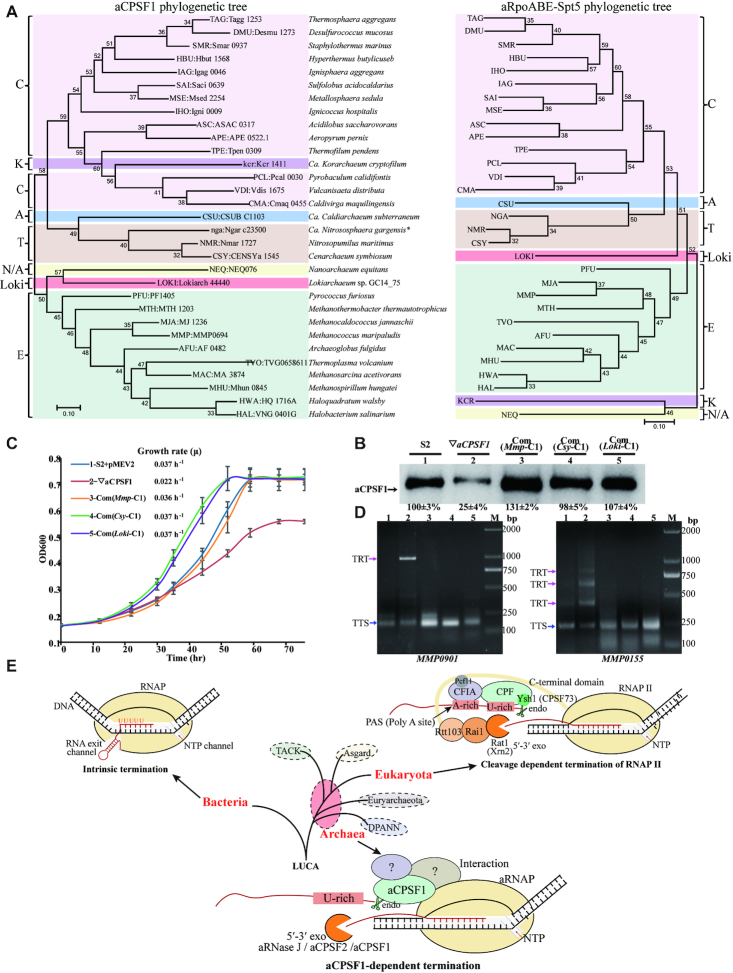
The ubiquitous distribution of the *aCPSF1* orthologs among Archaea and the aCPSF1 dependent archaeal transcription termination reveals a similar 3′-end cleavage mode as the eukaryotic RNAP II termination. (**A**) The Maximum Likelihood phylogenetic trees based on aCPSF1 proteins (left) and concatenated RNA polymerase subunits A, B and E, and transcription elongation factor Spt5 (right). Protein sequences were retrieved from genomes or metagenome-assembled genomes of the representative archaeal lineages that have been described thus far, including Euryarchaeota (E), Crenarchaeota (C), Thaumarchaeota (T), Aigarchaeota (A), Korarchaeota (K), Nanoarchaeota (N), and Lokiarchaeota (Loki). The unrooted tree was constructed using Maximum-Likelihood methods in MEGA 7.0 software package. Bootstrap values are shown at each node. (**B**) Protein abundances of the *aCPSF1* orthologs in complemented ▽*aCPSF1* strains were examined by western blot using the *Mmp*-aCPSF1 polyclonal antiserum. The percentiles of protein content referenced to that in S2 are shown beneath the gel. (**C**) Ectopic expression of the aCPSF1 orthologs from *Lokiarchaeum* GC14_75 (com(*Loki*-C1)) and *Cenarchaeum symbiosum* (com(*Csy*-C1)) restored the 22°C-growth defect of ▽*aCPSF1* similarly as the complementation of itself (com(*Mmp*-C1)) and wild-type S2 carrying an empty vector (S2-pMEV2). The averages of three replicates and standard deviations are shown. (**D**) 3′RACE assays the TRT transcripts of *MMP0901* and *MMP0155* in the strains numbered as in panel (B). (**E**) A proposed mechanism of aCPSF1 cleavage triggering the archaeal transcription termination exposes a homolog of the eukaryotic RNAP II termination mode (3–5). The bacterial intrinsic termination is included for comparison. LUCA means last universal common ancestor.

A previous phylogenomic analysis on the aCPSF1 orthologs retrieved from 110 complete archaeal genomes has indicated that this protein is strictly conserved and appear being inherited vertically (52). To explore the universality of aCPSF1-mediated transcription termination among Archaea, the aCPSF1 orthologs in 31 species representing all the four recently defined archaeal superphyla (Euryarchaeota, TACK, Asgard and DPANN) (56), were subjected to phylogenetic analysis in comparative with that of a protein concatenation of aRNAP subunits A, B and E, and the conserved transcription elongation factor Spt5 retrieved from the same species (Figure 6A). It was found that the aCPSF1 phylogeny exhibited an equivalent clustering topology as that of the concatenated sequences of 26 universally conserved proteins (57), and also a convergent clustering pattern as aRNAPs (Figure 6A). Thus, aCPSF1 appear displaying a similar conservative evolution as the RNA transcription machinery in Archaea.

Next, distribution of the terminator motif, uridine-rich sequence, was searched in each 200 nt upstream and downstream the putative stop codons of the annotated ORFs among 34 representative archaeal species. The result indicated that uridine-rich tracts with successive 5, 6 and 7 uridines (U5, U6 and U7) were significantly enriched in IGRs compared to the upstream gene-bodies in most searched archaeal genomes (Supplementary Figure S13 and Dataset S3). This indicates that the prevalent IGR uridine-rich tract could be a conserved terminator sequence of Archaea. The highly conservation of aCPSF1 and the prevalence of the 3′-end uridine-rich motif together suggest that the aCPSF1 dependent transcription termination could be universally employed in Archaea.

### Two phylogenetic distant aCPSF1 orthologs implement transcription termination in *M. maripaludis*

To verify if the aCPSF1 orthologs all function in transcription termination, two orthologs, one from Ca. *Lokiarchaeum* sp. GC14_75 belonging to Lokiarchaeota and the other from Ca. *Cenarchaeum symbiosum* affiliating with Thaumarchaeota, were selected. The two share 48% and 40% amino acid sequence identities with *Mmp*-aCPSF1 respectively (Supplementary Figure S14), and after codon optimized (Supplementary Figure S15), were ectopically expressed in ▽*aCPSF1* (Figure 6B). Behaving as *Mmp*-aCPSF1, the two orthologs not only restored the growth defect of ▽*aCPSF1* at 22°C (Figure 6C), but also eliminated the TRTs of convergent (*MMP0901*, type I) and co-directional TUs (*MMP0155*, type II) (Figure 6D). This demonstrated that the aCPSF1 orthologs from various archaeal phyla play a same role in transcription termination.

## DISCUSSION

Transcription termination mechanisms remain largely unknown in Archaea thus far (1,13,25), while very recently the ribonuclease aCPSF1, FttA, of *T. kodakarensis* was reported to function as a transcription terminate factor of Archaea mainly via an *in vitro* system (27). Almost at the same time, this work, through intensive genetic, molecular and biochemical experiments, and high-throughput RNA-seq and Term-seq, has revealed that the aCPSF1 ortholog of *M. maripaludis*, by cleaving at the transcript 3′-end uridine-rich sequences (Figure 4), triggers a genome-wide transcription termination (Figure 2), so guarantees the programmed transcriptome and an optimal growth as well as flagellation and motility (Figures 1 and 3) of the methanoarchaeon. aCPSF1, via associations with archaeal RNAP as well as the chromosome DNA (Figure 5), plays an essential role in archaeal genome-wide transcription termination. Therefore, aCPSF1 functions as a general transcription termination factor of Archaea, and the indispensable role in transcription termination reported here deciphers its universality and essentiality in Archaea.

Given the short IGRs distributed in the prokaryotic compact genomes, transcription termination could be more vital to an ordered transcriptome. This is verified by that an approximate 80% depleted expression of *Mmp*-*aCPSF1* caused a genome-wide TRT and disordered transcriptome with inversely changed expression of the highly- and poorly-transcribed genes (Figure 1E, F) and concomitant defective growth (Figure 1). This might be due to a deficient termination sequestering RNAP for new rounds of transcription. In addition, transcription readthrough particularly increases transcription of the downstream genes (type II TRT, 42%) (Figures 1 and 2), and has caused physiological changes, such as increased flagellation and cellular motility (Figure 3). This highlights the significance of aCPSF1 dependent transcription termination in maintaining an ordered transcriptome and optimal physiology of Archaea. Similar disordered transcriptomes derived from defective transcription termination are also found in Bacteria (58,59), in which the absence or depletion of the transcription termination factors caused genome-wide abnormal antisense, and intragenic and stable RNA transcriptions. In Eukaryotes, defective termination generates prevalent transcriptions of cryptic unstable RNA and the transcript 3′ flanking region, which disturbs ordered transcriptomes as well (4,8,60,61).

Term-seq analysis determines a terminator motif of two consecutive uridine-tracts in transcripts of *M. maripaludis* (Figure 4A). Similar, but slightly different, uridine-rich motifs have also been observed in three representative Euryarchaeota and Crenarchaeota species, *M.mazei*, *S.acidocaldarius* (13) and *H.volcanii* (24). Uridine-rich sequences were considered as the intrinsic termination signal based on the earlier and primarily *in vitro* studies (18,19,22), however, whether this sequence merely pauses or also dismantles aRNAP for termination has not been clarified. This work not only determined that aCPSF1 depletion causes an overall termination deficiency at the transcript 3′-end uridine-tract, but also demonstrated that *Mmp*-aCPSF1 performs an *in vitro* endoribonucleolytic cleavage at the putative TTSs downstream the uridine-rich sequences, and the *in vivo* endoribonuclease activity is determined to be essential to transcription termination (Figure 4). Thus, we suppose that both the uridine-rich termination signal and aCPSF1 are required by aRNAP for efficient transcription termination. Based on these results, a model shown in Figure 6E was proposed. The intrinsic uridine-rich termination signal directs the progressing aRNAP to pause or slowdown, which could buy time for aCPSF1 to recognize and cleave at downstream of the signal sequence. This event in turn triggers aRNAP dissociating from the DNA templates and nascent RNA, and results in transcription termination. An interaction with aRNAP (Figure 5A–D) warrants the recruitment of aCPSF1 to the transcription machinery and the uridine-rich motif in nascent RNAs. The high conservation of aCPSF1 (Figure 6A) and the prevalent uridine-rich motif in archaeal genome IGRs (Supplementary Figure S13 and Dataset S3) together suggest that the aCPSF1 mediated termination mechanism could be widely employed among Archaea. Supportively, two aCPSF1 orthologs from the distant related *Lokiarchaeota* and *Thaumarchaeota* were capable of implementing transcription termination in *M. maripaludis* (Figure 6B–D). Therefore, it is hypothesized that aCPSF1 dependent transcription termination could have an ancient origin predating the divergence of various archaeal phyla and is still widely employed in modern Archaea. Differently, based on a lower termination efficiency on cytidine-less than on guanine-less templates in an *in vitro* system, the *T. kodakarensis* aCPSF1 ortholog, FttA, was proposed to work on cytidine-rich sequences in a mode akin to the bacterial Rho protein (27). Whereas, Term-seq has revealed overrepresented uridine-rich sequences but not cytidine-rich upstream the transcript 3′-ends in four Archaea. Thus, the *in vivo* mechanism of FttA needs to be verified.

Remarkably, the working mechanism of aCPSF1 resembles the eukaryotic 3′-end processing/cleavage triggered RNAP II termination mode, in which the aCPSF1 homologs, the yeast Ysh1 or the human CPSF73, in the multi-subunits 3′-end processing machinery execute the 3′-end cleavage (3,5,8,62,63), and the accessory cleavage factors IA and IB (CFIA and CFIB) recognize the flanking uridine-rich sequences and facilitate the 3′-end cleavage of Ysh1 (62). However, aCPSF1 may rely on its N-terminal K homology (KH) domain, a characteristic RNA binding domain (52,53), to recognize the uridine-rich signals and implement the cleavage by itself. Interestingly, *Lokiarchaeota* that belongs to the Asgard superphylum, a supposed archaeal ancestor of Eukaryotes (15,64) appears using the aCPSF1 dependent termination mechanism as well. This is evidenced not only by that *Loki*-aCPSF1 is capable of terminating the methanococcal transcription (Figure 6B–D), but also the pervasive IGR uridine-rich sequences (Supplementary Dataset S3). Therefore, based on the homologous core components of the termination machineries, CPSF73 and aCPSF1, and the similar 3′-end cleavage triggered termination mechanisms, we proposed that the eukaryotic RNAP II termination might have evolved from the aCPSF1 dependent mode reported here, i.e. the aCPSF1 (CPSF73 homologous) mode exposed an archaeal precursory system of the eukaryotic transcription termination machinery. Distinctly, the eukaryotic CPSF73 cleavage downstream the 3′-end poly(A) site not only terminates transcription but also provides a polyadenylation site for transcript maturation (63,65), while no 3′ terminus polyadenylation follows aCPSF1 3′-end cleavage in Archaea. Moreover, very few homologs of the eukaryotic multi-subunit 3′-end processing complex (66,67) and termination factors (8–11) have been identified in Archaea, implying that Archaea could employ a simplified termination strategy. Additionally, the eukaryotic 5′-3′ exoribonuclease, like yeast Rat1 or human Xrn2, is involved in transcription termination by rapidly ‘chewing’ Ysh1/CPSF73 cleaved RNA products so to dismantle the associated RNAPs, noted as the ‘torpedo’ model (8,9,12,68). Although no homologs of Rat1 or Xrn2 have been found in Archaea, exoribonucleases aRNase J, aCPSF2 and even aCPSF1, all possessing the similar 5′-3′ exoribonucleolytic activities (43,51,52,69), could function comparatively in Archaea (Figure 6E). Therefore, the aCPSF1-dependent archaeal transcription termination could expose a mode akin to the eukaryotic RNAP II termination mechanism.

Interestingly, the 3′-end proximal uridine-rich tract likely represents a shared termination signal in the archaeal aCPSF1 termination system and the bacterial intrinsic termination (Figure 6E), as both could cause a pausing of the progressing RNAP and facilitate the upstream hairpin formation and aCPSF1 cleavage at nascent RNAs to trigger RNAP dissociation in bacteria and Archaea, respectively. Thus, compared to the simple intrinsic termination of bacteria, aCPSF1 and its eukaryotic homologs’ cleavage triggered terminations could expose complexity-upgraded transcription termination mechanisms in Archaea and Eukaryotes (Figure 6E), while the eukaryotic 3′ polyadenylation adds more complexity. Therefore, insight into aCPSF1 dependent archaeal transcription termination would shed light on understanding both the complex eukaryotic transcription termination and the evolutionary trajectory of life's transcription process. In conclusion, this work describes the first archaeal general transcription termination factor aCPSF1 and its cleavage dependent termination mechanism, which might be a precursory system of the eukaryotic RNAP II termination mode.

## DATA AVAILABILITY

Both the Term-seq and the strand-specific RNA-seq data have been deposited at the NCBI GEO Submission (GSE141346) [NCBI tracking system #20481779]. Custom codes used in this study are accessible at https://github.com/DXZbioinfor/aCPSF1-dependent-genome-wide-transcription-termination-of-Archaea.

## Supplementary Material

gkaa702_Supplemental_Files
