# Supplementary material for: The conserved ribonuclease aCPSF1 triggers genome-wide transcription termination of Archaea via a 3′-end cleavage mode
Source: Nucleic Acids Res. 2020 Aug 28;48(17):9589–605. doi: 10.1093/nar/gkaa702 (PMC7515710; doi:10.1093/nar/gkaa702)
Supplement: gkaa702_Supplemental_Files [file gkaa702_supplemental_files.zip › NAR-02037-H-2020-SM-R1.pdf]

Supplementary Information for

The conserved ribonuclease aCPSF1 triggers genome-wide transcription termination of Archaea via a 3′-end cleavage mode

Lei Yue[1,2,†], Jie Li[1, *†], Bing Zhang[3], Lei Qi[1], Zhihua Li[1,2,] Fangqing Zhao[3], Lingyan Li[1], Xiaowei Zheng[1], Xiuzhu Dong[1,2*]

1, State Key Laboratory of Microbial Resources, Institute of Microbiology, Chinese Academy of Sciences, Beijing 100101, PR China

2, University of Chinese Academy of Sciences, No.19A Yuquan Road, Shijingshan District, Beijing 100049, China

3, Beijing Institutes of Life Science, Chinese Academy of Sciences, Beijing 100101, China

*, Correspondence to Xiuzhu Dong, No.1 Beichen West Road, Beijing 100101. Tel. 86-10-6480 7413, Email: dongxz@im.ac.cn; Jie Li, No.1 Beichen West Road, Beijing 100101. Tel. 86-10-6480 7567. Email: lijie824@im.ac.cn.

†, these authors equally contributed.

This file contains:

Supplementary Figure. S1 to S15

Supplementary Tables S1 to S5

Caption for Supplementary Dataset S1: Defined transcriptional units (TUs) in the *M. maripaludis* S2 transcriptome and calculated transcriptional read-through (TRT).

Caption for Supplementary Dataset S2: Term-seq identified primary transcription termination sites (TTSs) in *M. maripaludis* S2.

Caption for Supplementary Dataset S3: Prevalence of the uridine-rich sequences in the IGRs among Archaea.

Caption for Supplementary Dataset S4: Differential transcription of *M. maripaludis* S2 vs *Mmp-aCPSF1* depletion mutant (∇*aCPSF1*).

Supplementary Figures

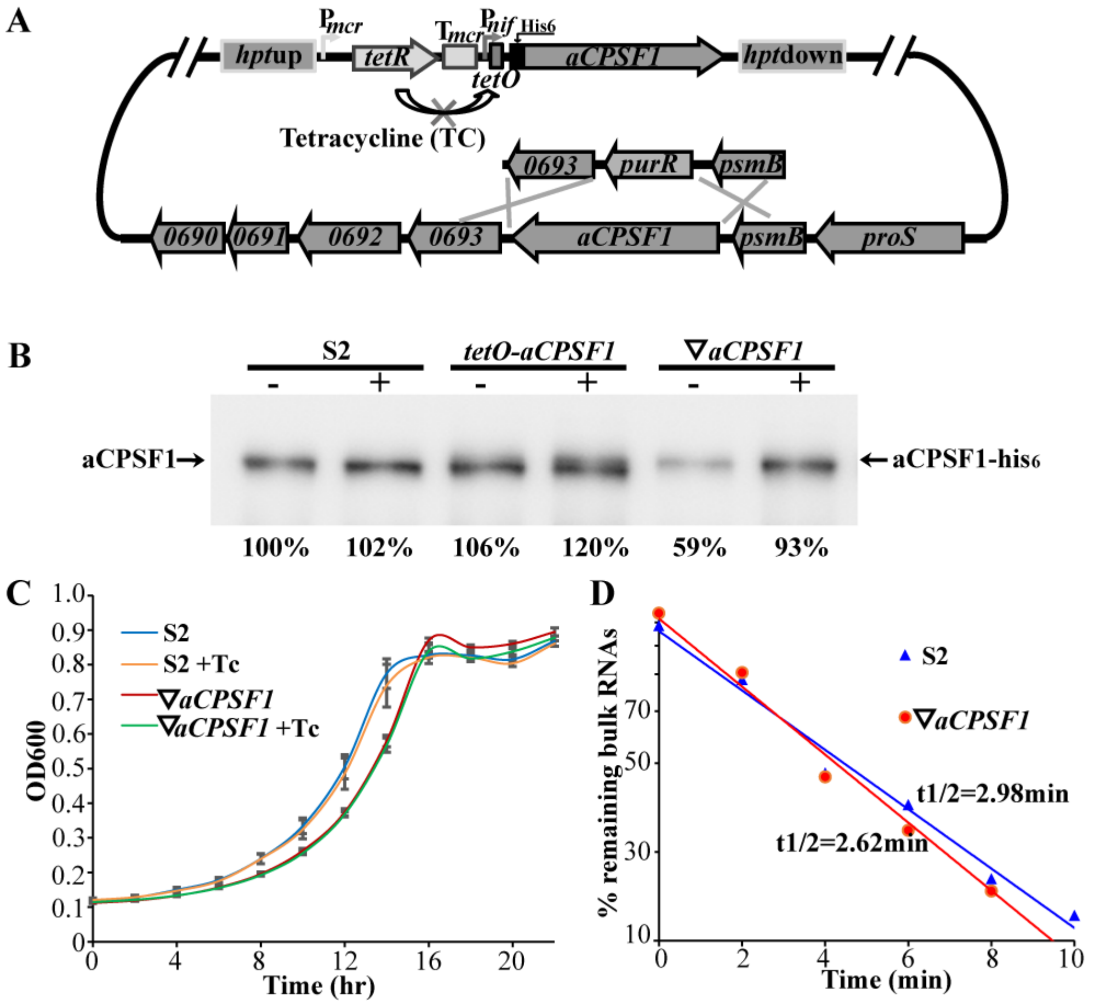

Figure S1. Effects of depleted expression of *Mmp-aCPSF1* on the 37°C-growth and cellular RNA lifespan of *M. maripaludis* S2. (*A*) A schematic depicts construction of the *Mmp-aCPSF1* (*MMP0694*) depletion strain (∇*aCPSF1*) (upper panel). The tetracycline (Tc) responsive regulator (*tetR*)-operator (*tetO*) cassette was fused to the His_6-tagged N-terminus of *Mmp-aCPSF1* and inserted into the *hpt* locus to obtain *Mmp-aCPSF1* ectopic expression strain (*tetO-aCPSF1*). Then the indigenous *Mmp-aCPSF1* gene was in-frame replaced by the puromycin resistance gene (*pur*^{R}) to result a TetR regulated *Mmp*-aCPSF1 depleted strain (∇*aCPSF1*). P*mcr* and T*mcr*, the promoter and terminator of the methoanococcal methyl-CoM reductase (*mcr*), respectively; P*nif*, the promoter of the methoanococcal *nif* gene. (***B***) Western blot detected the protein

abundance (percentages shown beneath by referenced to that of lane 1) of *Mmp*-aCPSF1 in the presence (+) or absence (–) of 100 μg/ml tetracycline (Tc) in 37°C-cultured S2 (wild-type), *tetO-aCPSF1*, and ▽*aCPSF1*. aCPSF1 and aCPSF1-His$_6$ flanked the gel pointed the indigenous and *hpt*-site inserted *Mmp*-aCPSF1, respectively. (***C, D***) *Mmp-aCPSF1* depletion slightly reduced the 37°C-growth (***C***) and increased the life-span of total mRNA (***D***). Half-lives of the total mRNA are calculated from the regression curve of residual mRNA by quantification of the [^{3}H]-uridine signal attenuation as described in Methods. Experiments were performed on three batches of culture, averages and standard deviations were shown.

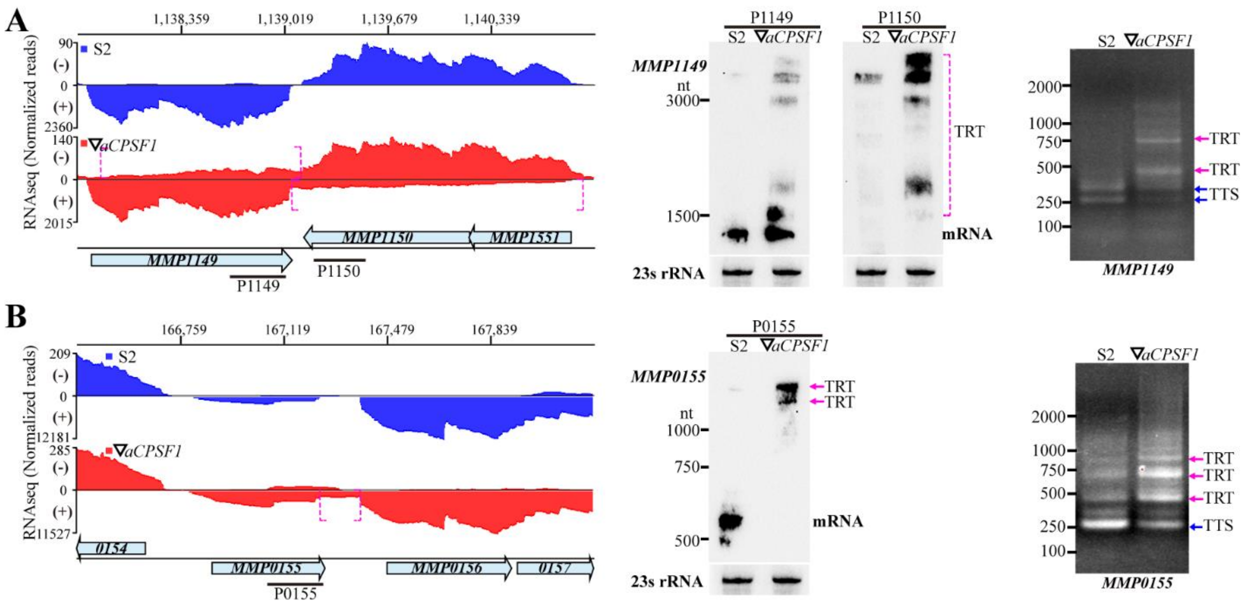

Figure S2. Northern blot and 3′RACE verifications of RNA-seq detected TRTs in the *Mmp-aCPSF1* depletion mutant. Similar to Fig. 2*A*, *MMP1149*, encoding 3-isopropylmalate dehydratase, and the antisense *MMP1150* for uroporphyrinogen decarboxylase (***A***), and *MMP0155* encoding a putative RNA-binding protein (***B***) that exhibited 3′-end extension (bracketed with magenta line) upon *Mmp-aCPSF1* depletion were selected for verification. Left panels show the RNA-seq reads mapped to the corresponding genes in strains S2 (blue) and ∇*aCPSF1* (red). Numbers on the top indicate nucleotide sites of the mapped genomic regions, and bullets represent gene orientations. Middle panels show Northern blot detected transcripts with normal termination (TTS) and TRT (magenta arrows or frame). Right panels show 3′RACE amplified products with normal TTS (blue arrows) and TRT (magenta arrows) in S2 and ∇*aCPSF1*, respectively. The sequences of 3′RACE assayed 3′-end nucleotides are shown in Fig. S6.

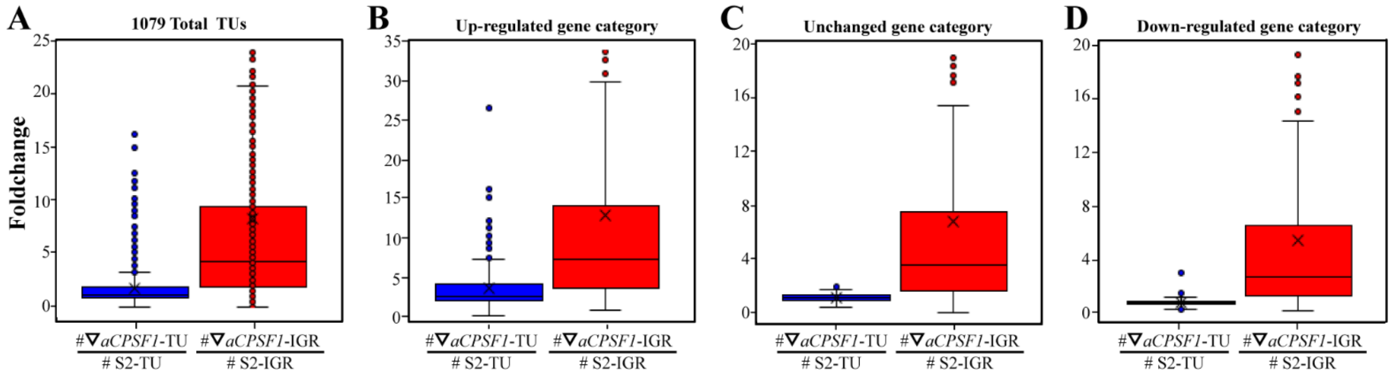

Figure S3. *Mmp-aCPSF1* depletion causes higher transcription increases in IGR than in the TU body. Boxplots show the FPKM fold changes of 1,079 TUs and the flanking IGRs in ▽*aCPSF1* compared to that of S2 (***A***). Based on the transcription changes upon *Mmp-aCPSF1* depletion, these TUs are further classified into three categories as Up-regulated (***B***), Unchanged (***C***), and Down-regulated (***D***). Boxplots show the FPKM fold change statistics in IGRs and the associated TU bodies, and the fold change values of 50% genes are shown inside the box and the medians are shown as lines inside the boxes.

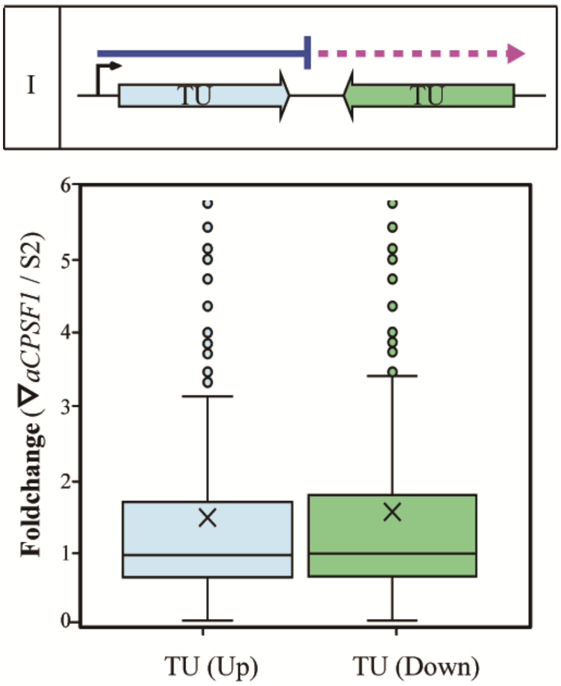

Figure S4. Transcription abundance changes of the convergent TUs in ∇*aCPSF1* and S2 strains. The upper panel shows the schematic of the convergent TUs. Bent arrows and horizontal blue lines indicate TSS and transcript lengths in strain S2, respectively, and dot magenta arrows indicate transcripts that occur TRT in ∇*aCPSF1*. Boxplots (lower panel) show the FPKM fold changes of 496 convergent TUs in ∇*aCPSF1* compared to that in S2. The fold change values of 50% genes are shown inside the box and the medians are shown as lines inside the boxes.

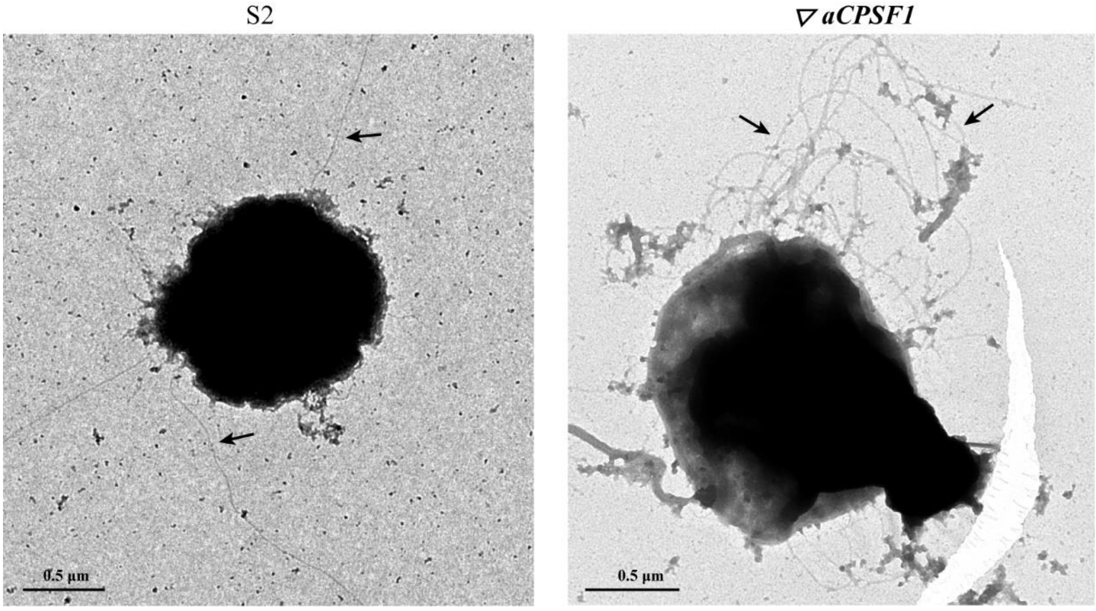

Figure S5. Electron microscopic images display more archaella in 22°C-grown ∇*aCPSF1* than in S2 cells. Arrows indicate the archaella.

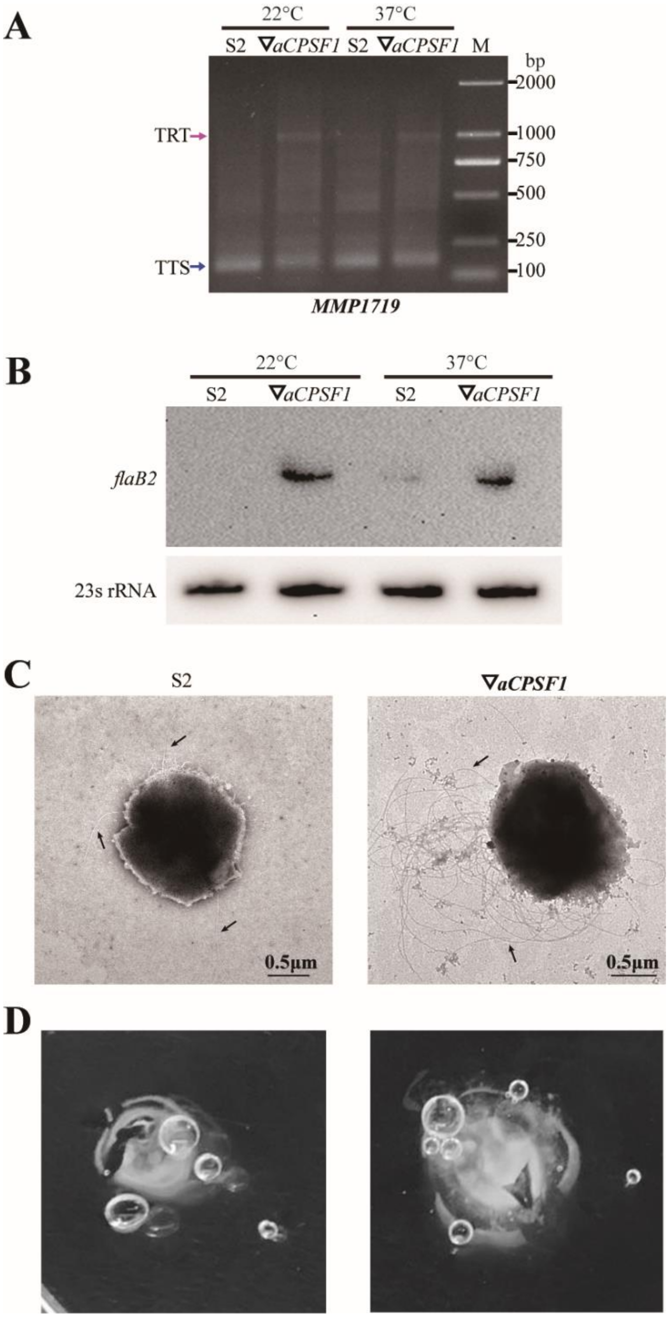

Figure S6. *MMP1719* TRT upregulates the transcription of the *fla* operon genes and results in exuberant archaella along with elevated motility in 37°C-cultured ∇*aCPSF1*. (***A***) 3′RACE amplified fragments contain the transcription termination site (TTS) in S2 and TRT of *MMP1719* in ∇*aCPSF1* at the 22°C and 37°C cultures, respectively. (***B***) Northern blot assayed the *flaB2* transcript abundances in 22°C- and 37°C-cultured strains S2, and ∇*aCPSF1*. 23S rRNA was used as the sample control. (***C***) Representative electron microscopic images show more archaella (arrows pointed)

at 37°C-cultured ▽*aCPSF1* than S2 cells. (***D***) Mobility assay displays larger lawn in 37°C-cultured ▽*aCPSF1* than that in S2 when grown on 0.25% agar.

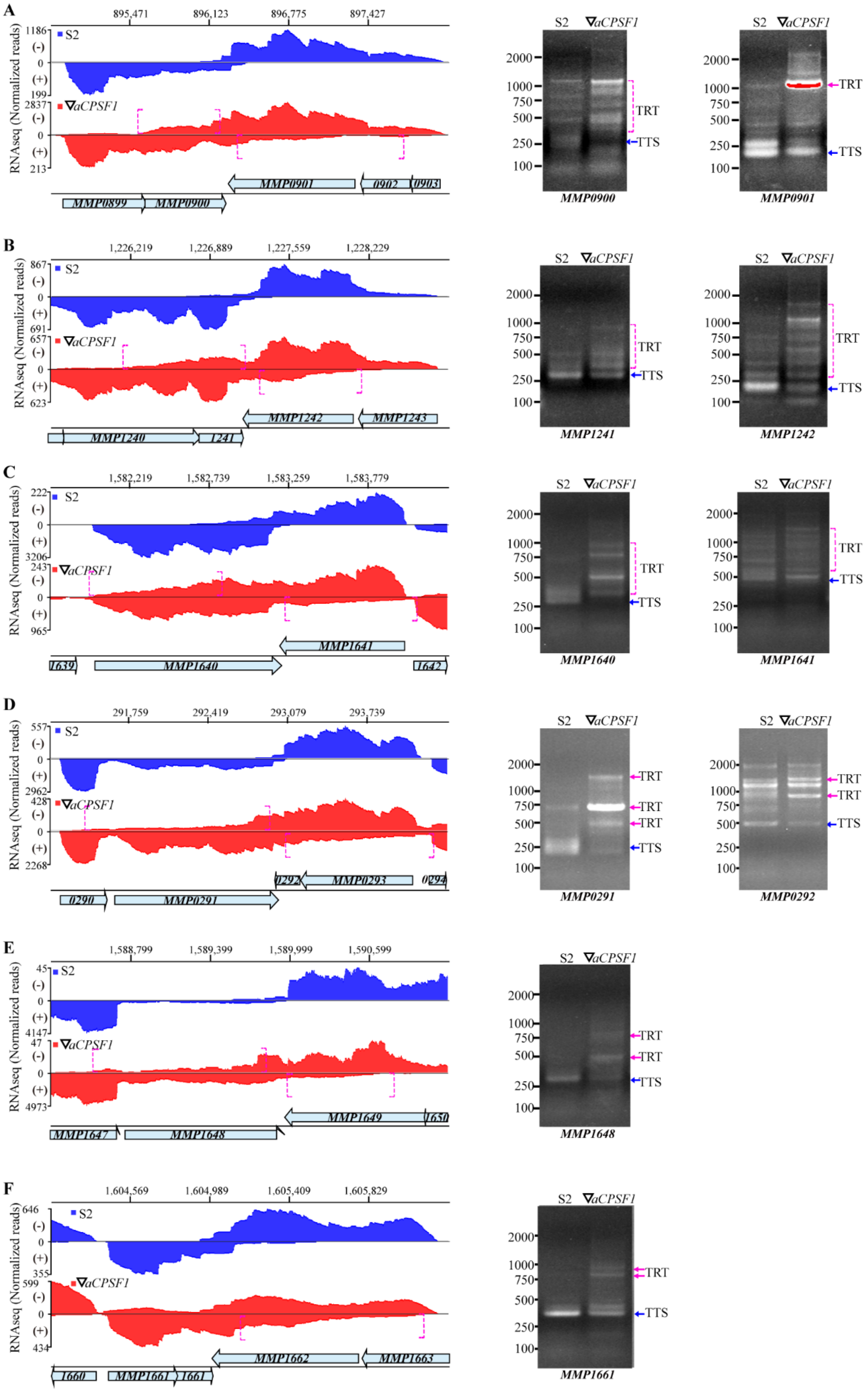
A
B
C
D
E
F
S2
∇aCPSF1
RNAseq (Normalized reads)
TRT
TTS
MMP0900
MMP0901
MMP1241
MMP1242
MMP1640
MMP1641
MMP0291
MMP0292
MMP1648
MMP1661

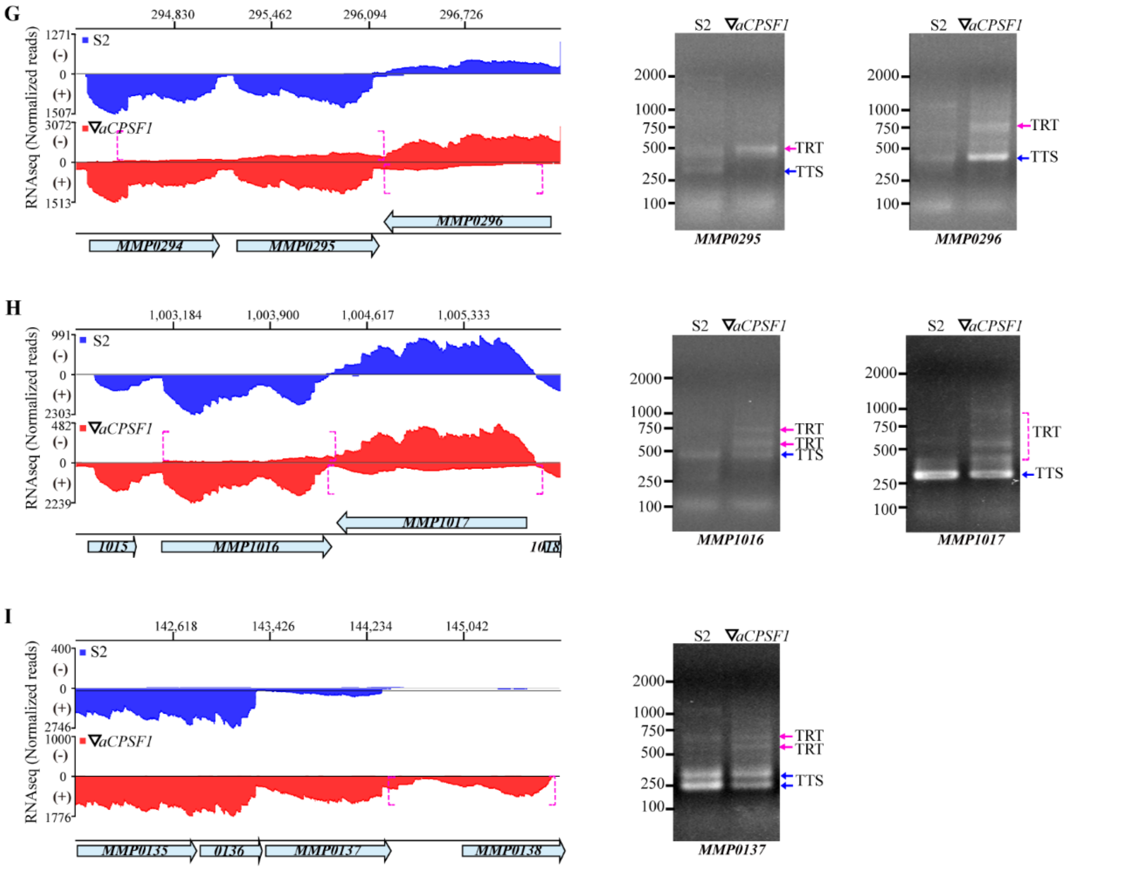

Figure S7. 3′RACE validation of *Mmp-aCPSF1* depletion caused TRTs in fifteen genes. Using the same approach as in Fig. 2*A* and Fig. S3, additional fifteen representative transcripts that exhibited 3′-end extension (dotted magenta brackets) in ∇*aCPSF1* were selected for experimental validation. *MMP0900* and *MMP0901* encode tRNA 2-selenouridine synthase and a small GTP-binding protein, respectively (*A*); *MMP1241* and *MMP1242* encode a hypothetical protein and quinolinate synthetase subunit A, respectively (***B***); *MMP1640* and *MMP1641* encode S-adenosylmethionine synthetase and soluble P-type ATPase (***C***); *MMP0291, MMP0292* (***D***)*, MMP1648, MMP1449* (***E***), *MMP1661* and *MMP1663* (***F***) all encode hypothetical proteins; *MMP0295* and *MMP0296* encode homoserine kinase and nonsense-mediated mRNA decay protein (***G***); *MMP1016* and MMP1017 encode a hypothetical protein and aspartate kinase, respectively (***H***); and *MMP0137* encodes deoxyhypusine synthase (***I***).

Left panels show the RNA-seq reads mapped to the corresponding genes in strains S2 (blue) and ▽*aCPSF1* (red), respectively. Numbers on the top indicate the nucleotide sites of mapped genomic regions, and bullets represent gene orientations. Right panels show 3′RACE amplified products with natural termination (TTS, blue arrows) and TRT (magenta arrows) of the indicated genes beneath the gel. The sequences of 3′RACE assayed nucleotides at the transcript 3′-ends are shown in Supplementary Figure S8.

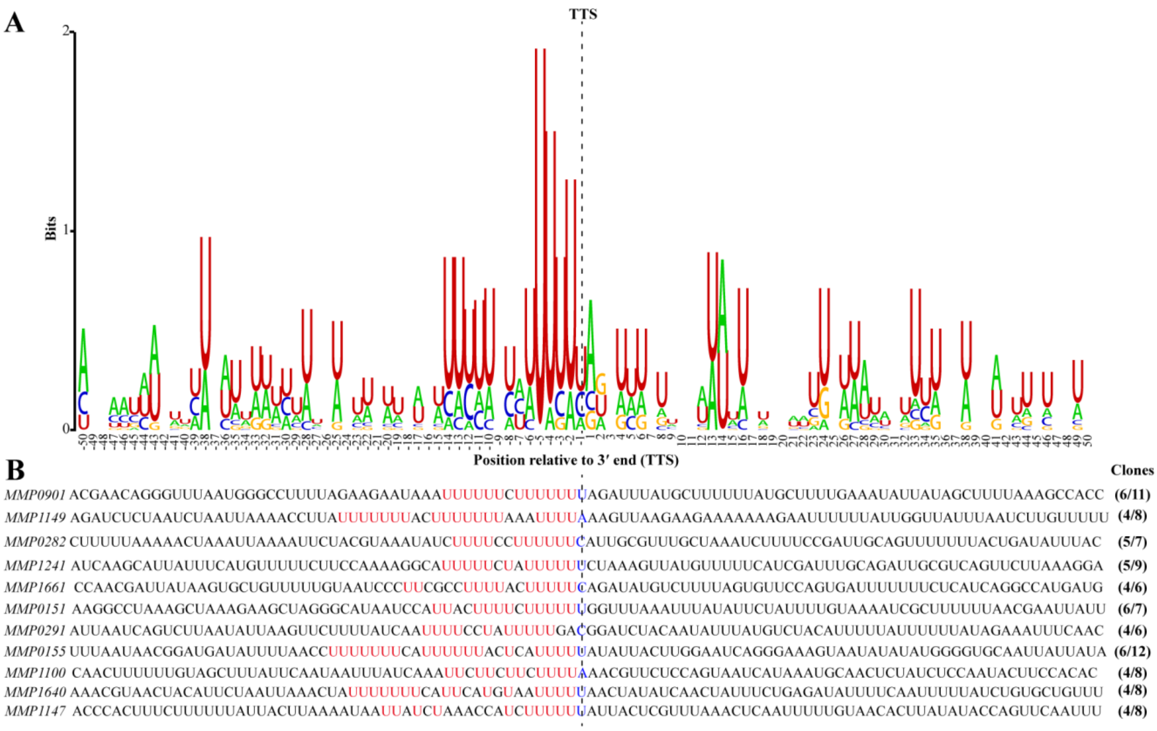

Figure S8. The terminator motifs determined by 3′RACE. Using the WebLogo, a terminator motif of uridine-rich tract (***A***) was generated according to the 3′RACE captured 3′-end sequence (TTS, dot line) among 11 transcription units (***B***). Similar terminator motif was also found in the Term-seq data (Fig. 4*A*). Sequences in length of 50 nt each upstream and downstream the Term-seq detected TTSs of the 11 TUs are shown in (***B***), in which the uridine-rich stretches are in red. Inside parenthesis indicate the sequenced clone numbers of 3′RACE amplified fragments, and the former indicates the clone numbers matching the Term-seq determined TTS.

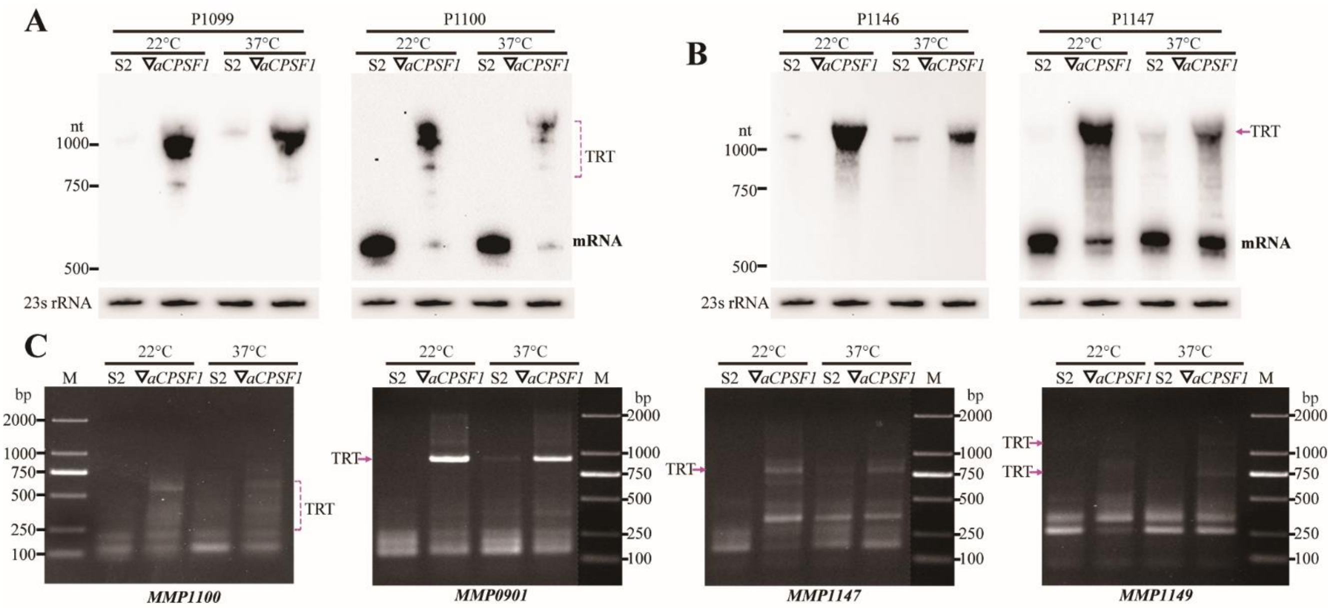

Figure S9. Northern blot and 3′RACE validation of *Mmp-aCPSF1* depletion caused TRTs in the 37°C cultures. (*A, B*) Northern blot assayed TRTs and transcript abundance changes of the TU pairs *MMP1099-MMP1100* and *MMP1147-MMP1146* in 37°C-cultured ▽*aCPSF1* compared to S2 cells. (***C***) 3′RACE verified the TRTs of *MMP1100, MMP0901, MMP1147* and *MMP1149* in 37°C-cultured ▽*aCPSF1* compared to S2 cells. The 22°C-cultured ▽*aCPSF1* and S2 samples were used as controls.

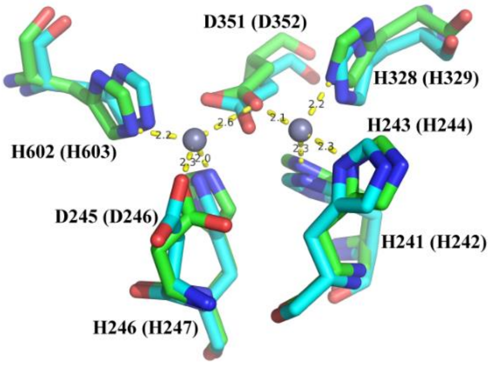

Figure S10. The predicted catalytic center of *Mmp*-aCPSF1. Using mm_0695 (PDB ID: 2XR1), an aCPSF1 from *Methanosarcina mazei* as template, the protein structure of *Mmp*-aCPSF1 (MMP0694) was homology-modelled using SWISS-MODEL (https://swissmodel.expasy.org/). The catalytic center with two zinc ions (light blue spheres) and seven conserved residues (sticks of green for mm_0695 and cyan for MMP0694) is shown. Inside the parentheses indicate the residue numbers of *Mmp*-aCPSF1. Dot yellow lines show the predicted hydrogen-bond interactions between zinc ions and residues, and numbers at the lines indicate hydrogen-bond length in Å.

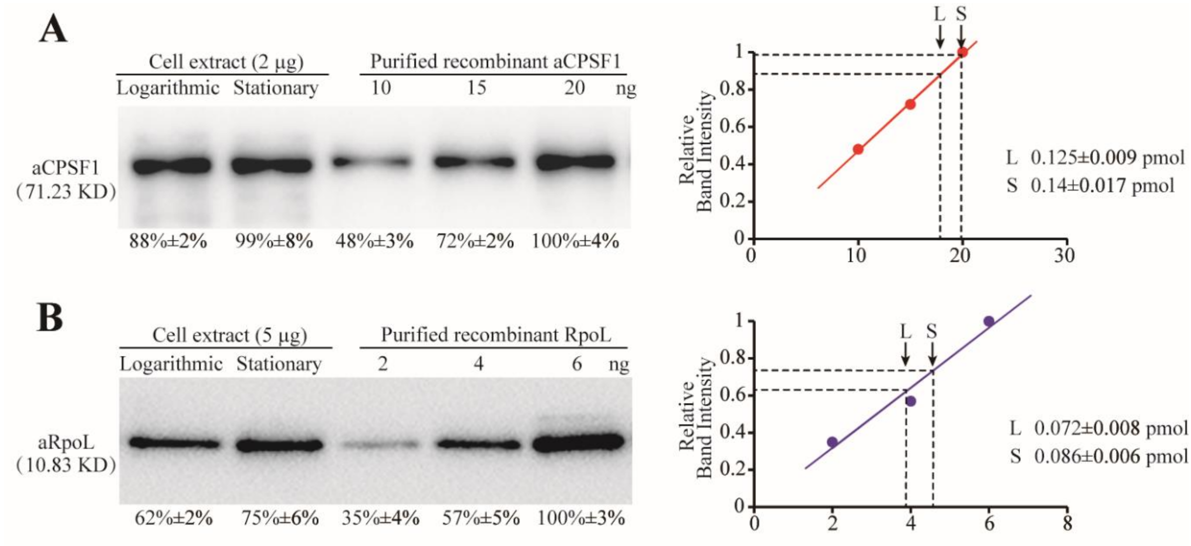

Figure S11. The cellular protein abundances of aCPSF1 and RNA polymerase determined by quantitative western blot. The cell extract and purified recombinant aCPSF1 and aRpoL, one of the RNA polymerase subunits, are loaded on SDS-PAGE gel with indicated contents. Protein contents of aCPSF1 (***A***) and aRpoL (***B***) were then assayed via western blot using respective antibodies (left). The cellular aCPSF1 and aRpoL contents (pmol) are determined at logarithmic (L, OD600=0.6) and stationary (S, OD600=0.9) phases per μg total cell proteins, and the protein molecules are calculated based on the calibrations (right).

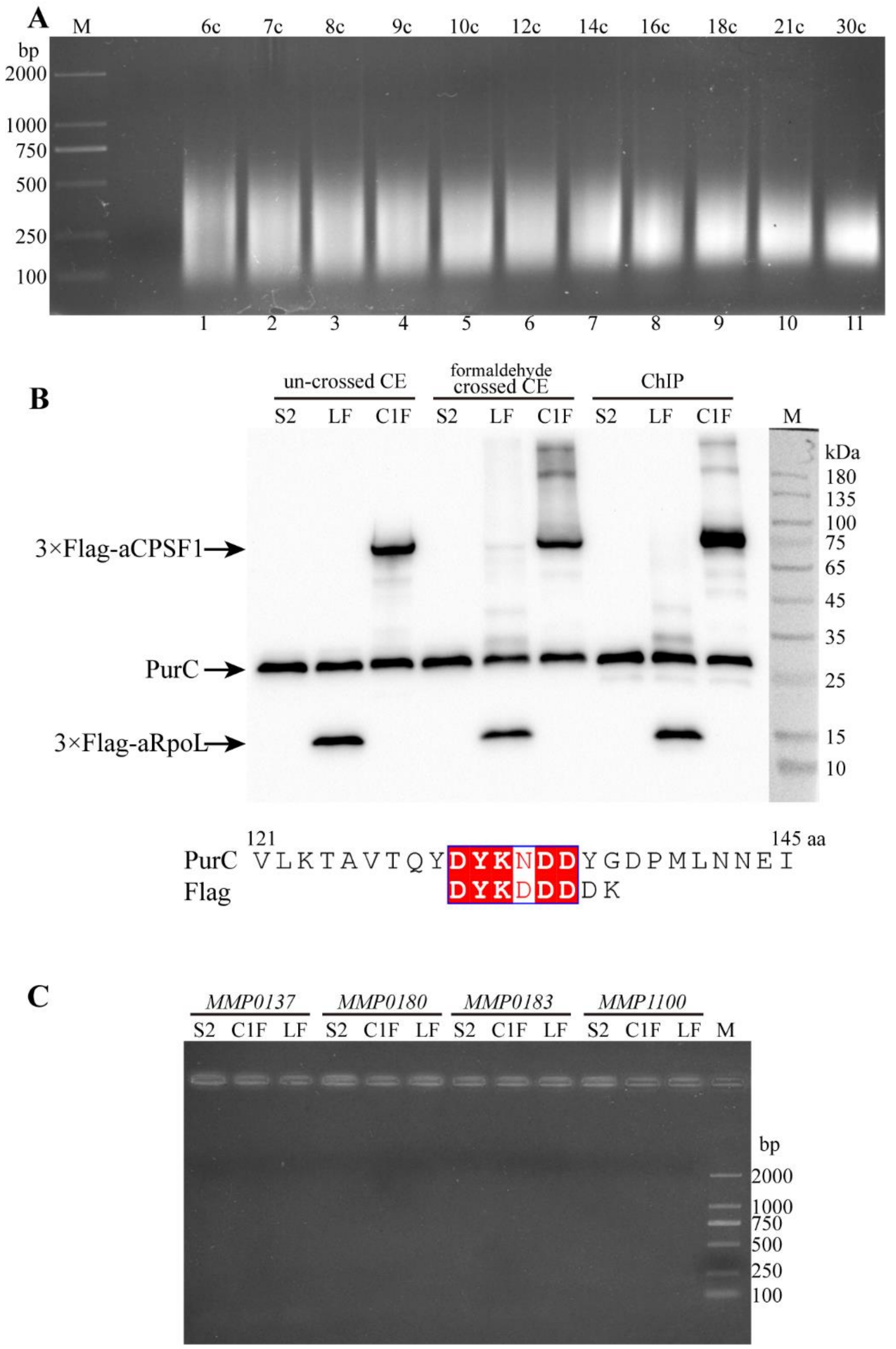

Figure S12. Examinations of the sheared chromosomal DNA and the tagged proteins used in ChIP assays. (***A***) Sonication sheared DNA fragments of the chromosomal DNA are detected by agarose gel. Bioruptor UCD300 (Diagenode) was used through various sonication cycles (lanes 1–11) as indicated on the top of gel. M, DNA ladders to indicate the migration position. The suffix c indicates the sonication cycles. (***B***) Western blot detected 3×Flag-aCPSF1 and 3×Flag-aRpoL in un-crossed cell

extracts (CE), the formaldehyde crossed CE and ChIP samples in strains S2 (mock), *Mmp*-RpoL-HF (LF) and *Mmp*-aCPSF1-F (C1F). Formaldehyde crossed CE also indicates the ChIP input. The detected PurC band was assumed due to the recognition of anti-Flag antibody (1) to PurC protein since a sequence similarity between PurC (130-135 amino acids) and Flag as shown in lower panel. (***C***) The sequences downstream the TTSs of *MMP0137*, *MMP0180*, *MMP0183* and *MMP1100* were PCR amplified as the negative control of that in Figure 5D. The ChIP DNAs of strains S2 (mock), *Mmp*-RpoL-HF (LF), and *Mmp*-aCPSF1-F (C1F) were used as the PCR templates, respectively. M, DNA marker.

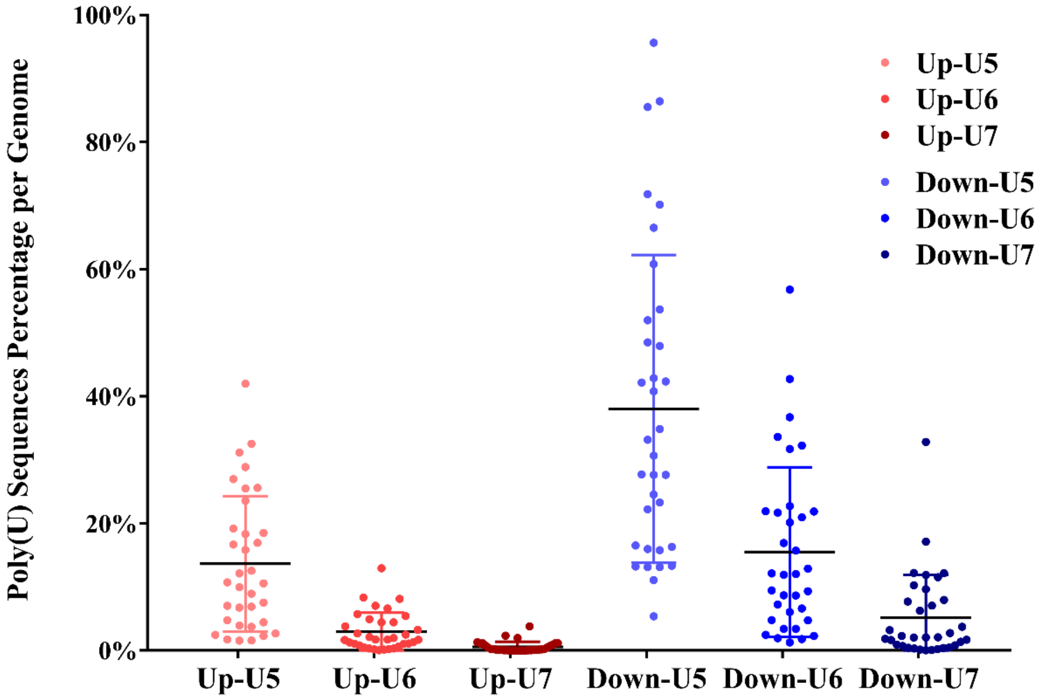

Figure S13. Enrichment of the uridine-rich sequences in the archaeal IGRs. The uridine-rich tract (U5, U6 and U7) sequences were searched at each 200 nt of upstream (Up) and downstream (Down) of the predicted stop codons in the annotated ORFs (Dataset S4) from representative species of the four defined archaeal superphyla. The poly(U) sequence percentage was calculated by the uridine-rich tract numbers over the gene numbers in each selected genome. Black lines refer to the percentage medians and the colored lines indicate the lower to the upper quartile, respectively. U5, U6 and U7 indicate 5, 6 and 7 successive uridines, respectively. A T-Test statistical analysis has been performed for distribution significance tests of the three U-rich tracts between up- and downstream ORFs, and the P-values for U5, U6 and U7 tracts are 3.81E-11, 1.28E-7 and 0.00013 respectively.

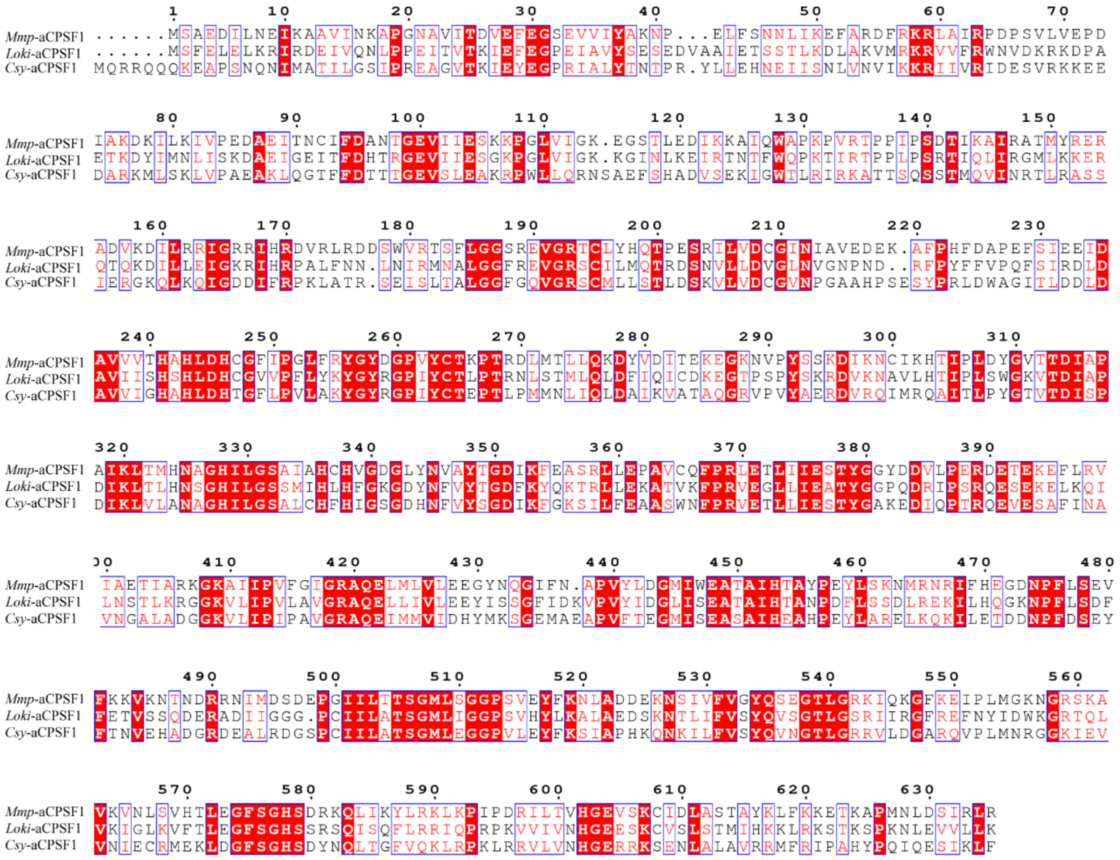

Figure S14. Sequence alignment between *Mmp*-aCPSF1 and *Loki*-aCPSF1 and *Csy*-aCPSF1. Sequences were aligned using ClustalW program(2) and the diagram was prepared using ESPript program(3). Identical residues are highlighted with white type on a red background and similar residues are shown as red type. *Mmp*-aCPSF1 shares 48% and 40% amino acid sequence identities and 69% and 60% amino acid sequence similarities to *Loki*-aCPSF1 from *Ca. Lokiarchaeum* GC14_75 and *Csy*-aCPSF1 from *Ca. Cenarchaeum symbiosum,* respectively.

```
Lokiarch_44440 M  S  F  E  L  E  L  K  R  I  R  D  E  I  V  Q  N  L  P  P  E  I  T  V  T  K  I  E  F  E
Original 1     ATGAGTTTTGAATTAGAACTTAAAAGAATTAGAGACGAAATTGTCCAGAATCTACCCCCCGAAATTACTGTTACAAAAATCGAATTTGAG
Optimized      ATGTCATTTGAATTAGAACTTAAAAGAATTAGAGATGAAATAGTTCAAAATTTGCCACCAGAAATAACAGTTACTAAAATAGAATTTGAA
Lokiarch_44440 G  P  E  I  A  V  Y  S  E  S  E  D  V  A  A  I  E  T  S  S  T  L  K  D  L  A  K  V  M  R
Original_91    GGTCCAGAAATTGCTGTTTATTCAGAGAGTGAGGATGTTGCCGCTATTGAAACCTCTTCTACCTTAAAAGATTTAGCGAAGGTGATGAGA
Optimized      GGACCAGAAATTGCAGTTTATTCAGAAAGTGAAGATGTTGCAGCAATTGAAACTTCATCTACTTTAAAGGATCTTGCAAAAGTTATGAGA
Lokiarch_44440 K  R  V  V  F  R  W  N  V  D  K  R  K  D  P  A  E  T  K  D  Y  I  M  N  L  I  S  K  D  A
Original_181   AAAAGAGTTGTGTTTAGGTGGAACGTAGATAAACGCAAAGACCCAGCAGAAACTAAAGACTATATAATGAACCTAATCAGCAAAGATGCT
Optimized      AAAAGAGTAGTATTCAGATGGAATGTTGATAAAAGGAAAGATCCTGCTGAAACTAAAGATTACATTATGAATCTTATTTCAAAAGATGCA
Lokiarch_44440 E  I  G  E  I  T  F  D  H  T  R  G  E  V  I  I  E  S  G  K  P  G  L  V  I  G  K  K  G  I
Original_271   GAAATTGGCGAAATAACCTTCGATCACACCCGTGGCGAAGTTATCATCGAATCAGGAAAACCAGGACTAGTAATAGGTAAAAAAGGAATT
Optimized      GAAATAGGTGAAATAACTTTTGATCACACTAGAGGTGAAGTAATTATTGAATCAGGAAAACCAGGATTAGTAATTGGTAAAAAAGGTATA
Lokiarch_44440 N  L  K  E  I  R  T  N  T  F  W  Q  P  K  T  I  R  T  P  P  L  P  S  R  T  I  Q  L  I  R
Original_361   AATTTAAAAGAAATTAGAACAAACACATTCTGGCAGCCAAAAACTATCAGAACACCACCTCTACCTTCTAGAACAATTCAATTAATTAGA
Optimized      AATCTTAAAGAAATTAGAACAAATACATTTTGGCAGCCTAAAACAATAAGAACCCCACCTTTACCTTCTAGGACTATTCAGTTAATAAGA
Lokiarch_44440 G  M  L  K  K  E  R  Q  T  Q  K  D  I  L  L  E  I  G  K  R  I  H  R  P  A  L  F  N  N  L
Original_451   GGTATGCTTAAAAAAGAAAGACAAACACAGAAAGACATTCTTTTAGAAATAGGAAAAAGAATTCACAGACCTGCACTATTTAACAATTTA
Optimized      GGAATGCTTAAAAAAGAAAGACAAACACAGAAAGATATTCTTTTAGAAATAGGTAAAAGAATTCATAGACCAGCATTATTCAATAACTTA
Lokiarch_44440 N  I  R  M  N  A  L  G  G  F  R  E  V  G  R  S  C  I  L  M  Q  T  R  D  S  N  V  L  L  D
Original_541   AATATAAGAATGAATGCATTAGGGGGTTTTCGCGAAGTTGGGCGATCCTGTATATTAATGCAGACGAGAGATAGTAATGTTTTACTAGAC
Optimized      AATATTAGAATGAATGCTTTAGGAGGTTTTAGAGAAGTAGGTAGATCATGCATTTTAATGCAGACTAGAGATTCAAATGTTTTATTAGAT
Lokiarch_44440 V  G  L  N  V  G  N  P  N  D  R  F  P  Y  F  F  V  P  Q  F  S  I  R  D  L  D  A  V  I  I
Original_631   GTGGGATTGAATGTAGGGAATCCGAATGACAGGTTTCCTTATTTCTTTGTCCCACAATTTTCCATTCGTGATCTTGATGCTGTAATCATT
Optimized      GTAGGTCTTAATGTTGGAAATCCAAATGATAGATTTCCATACTTTTTCGTTCCTCAATTTTCTATTAGGGATCTTGATGCAGTAATAATT
Lokiarch_44440 S  H  S  H  L  D  H  C  G  V  V  P  F  L  Y  K  Y  G  Y  R  G  P  I  Y  C  T  L  P  T  R
Original_721   TCACACTCGCACCTTGATCATTGTGGTGTTGTTCCCTTTCTATATAAATATGGTTATCGTGGTCCAATTTATTGTACATTACCTACAAGA
Optimized      AGTCATTCTCACCTTGATCACTGCGGAGTTGTTCCATTTTTATATAAATATGGTTATAGAGGACCAATCTATTGTACACTTCCAACAAGA
Lokiarch_44440 N  L  S  T  M  L  Q  L  D  F  I  Q  I  C  D  K  E  G  T  P  S  P  Y  S  K  R  D  V  K  N
Original_811   AATTTATCAACAATGTTACAGCTTGATTTCATTCAAATTTGTGACAAAGAAGGAACTCCATCACCATATTCAAAAAGAGATGTTAAAAAT
Optimized      AACCTTTCAACAATGTTACAGTTAGATTTTATTCAAATATGTGATAAAGAAGGAACTCCAAGTCCATATAGTAAAAGAGATGTTAAAAAT
Lokiarch_44440 A  V  L  H  T  I  P  L  S  W  G  K  V  T  D  I  A  P  D  I  K  L  T  L  H  N  S  G  H  I
Original_901   GCAGTACTTCATACAATTCCCCTCTCTTGGGGTAAAGTTACTGACATTGCTCCGGATATAAAACTTACACTTCATAATTCGGGTCATATT
Optimized      GCAGTATTACATACCATACCATTAAGTTGGGGTAAAGTTACAGATATTGCTCCTGATATAAAATTAACATTACATAATAGTGGACACATT
Lokiarch_44440 L  G  S  S  M  I  H  L  H  F  G  K  G  D  Y  N  F  V  Y  T  G  D  F  K  Y  Q  K  T  R  L
Original_991   TTAGGATCTTCTATGATACATTTACATTTTGGGAAGGGAGATTATAATTTTGTGTATACAGGGGATTTAAATATCAAAAAACTCGATTG
Optimized      TTGGGTTCATCAATGATTCACTTACACTTTGGTAAAGGAGATTATAATTTTGTTTACACAGGAGATTTTAAATACCAAAAAACAAGGTTA
Lokiarch_44440 L  E  K  A  T  V  K  F  P  R  V  E  G  L  L  I  E  A  T  Y  G  G  P  Q  D  R  I  P  S  R
Original_1081  CTAGAAAAAGCCACAGTTAAATTTCCAAGAGTTGAAGGCCTTTTAATTGAAGCTACTTATGGTGGGCCTCAAGATCGCATCCCAAGCAGA
Optimized      TTAGAAAAAGCAACTGTAAAATTTCCAAGAGTTGAAGGACTTTTAATAGAAGCAACATATGGTGGTCCTCAAGATAGAATTCCTTCTAGA
Lokiarch_44440 Q  E  S  E  K  E  L  K  Q  I  L  N  S  T  L  K  R  G  G  K  V  L  I  P  V  L  A  V  G  R
Original_1171  CAAGAATCTGAAAAGGAGTTAAAACAGATTTTAAATTCCACACTCAAAAGAGGTGGAAAAGTTCTAATACCTGTTCTGGCTGTAGGACGT
Optimized      CAAGAATCTGAAAAAGAACTCAAACAAATTCTTAATTCAACTCTTAAAAGAGGTGGAAAAGTTTTAATTCCAGTATTAGCAGTAGGAAGA
Lokiarch_44440 A  Q  E  L  L  I  V  L  E  E  Y  I  S  S  G  F  I  D  K  V  P  V  Y  I  D  G  L  I  S  E
Original_1261  GCTCAGGAATTATTAATTGTTTTAGAAGAATACATCTCATCAGGATTTATTGATAAAGTACCAGTCTATATTGATGGTCTAATAAGTGAA
Optimized      GCTCAGGAATTATTAATTGTTTTAGAAGAATATATTTCATCAGGTTTTATTGATAAAGTTCCAGTTTATATTGATGGATTAATTTCAGAA
Lokiarch_44440 A  T  A  I  H  T  A  N  P  D  F  L  S  S  D  L  R  E  K  I  L  H  Q  G  K  N  P  F  L  S
Original_1351  GCCACCGCAATCCACACGGCCAATCCTGATTTTCTGAGCAGTGATTTAAGAGAAAAAATACTTCATCAAGGGAAAAATCCATTTTTAAGC
Optimized      GCAACAGCAATTCATACGGCTAATCCTGACTTTTTAAGTTCTGACTTAAGAGAAAAAATATTACACCAAGGAAAAAATCCATTTTTAAGT
Lokiarch_44440 D  F  F  E  T  V  S  S  Q  D  E  R  A  D  I  I  G  G  G  P  C  I  I  L  A  T  S  G  M  L
Original_1441  GACTTTTTTGAGACCGTTTCATCTCAAGATGAGAGAGCTGACATAATTGGGGGAGGTCCATGTATAATACTAGCTACTAGTGGCATGCTA
Optimized      GATTTCTTTGAAACTGTTTCTTCTCAGGATGAAAGAGCAGATATTATAGGAGGAGGACCATGTATAATTTTAGCAACATCAGGAATGTTG
Lokiarch_44440 I  G  G  P  S  V  H  Y  L  K  A  L  A  E  D  S  K  N  T  L  I  F  V  S  Y  Q  V  S  G  T
Original_1531  ATTGGAGGACCTTCAGTTCATTACTTAAAAGCTCTAGCAGAGGATTCAAAAAACACATTGATTTTCGTATCTTATCAAGTTTCAGGGACT
Optimized      ATAGGAGGACCTAGTGTTCATTACTTAAAAGCTTTAGCAGAAGATTCAAAAAATACATTAATTTTTGTAAGTTACCAAGTTTCAGGAACA
Lokiarch_44440 L  G  S  R  I  I  R  G  F  R  E  F  N  Y  I  D  W  K  G  R  T  Q  L  V  K  I  G  L  K  V
Original_1621  TTAGGAAGCAGGATAATTCGTGGGTTTAGAGAATTCAATTATATAGATTGGAAAGGTCGAACACAGCTAGTAAAAATAGGTTTAAAAGTA
Optimized      TTAGGATCTAGAATAATAAGAGGTTTTAGAGAATTCAATTACATTGATTGGAAAGGAAGAACACAGTTGGTTAAAATAGGACTTAAAGTA
Lokiarch_44440 F  T  L  E  G  F  S  G  H  S  S  R  S  Q  I  S  Q  F  L  R  R  I  Q  P  R  P  K  V  V  I
Original_1711  TTTACTCTTGAAGGTTTTAGTGGACATTCATCTCGTAGCCAAATTTCTCAATTTCTGAGAAGAATTCAGCCAAGACCAAAGGTAGTTATA
Optimized      TTCACACTTGAAGGATTTTCAGGACACTCATCAAGATCACAGATTTCACAGTTTTTAAGAAGAATTCAGCCTAGACCTAAAGTTGTAATT
Lokiarch_44440 V  N  H  G  E  E  S  K  C  V  S  L  S  T  M  I  H  K  K  L  R  K  S  T  K  S  P  K  N  L
Original_1801  GTAAATCATGGAGAAGAAAGTAAATGCGTAAGTTTATCAACAATGATACATAAAAAGCTAAGGAAATCAACTAAAAGTCCTAAAAATCTT
Optimized      GTTAATCACGGAGAAGAATCTAAATGTGTATCTTTAAGTACAATGATTCATAAAAAATTAAGAAAAAGTACTAAATCACCTAAAAACTTA
Lokiarch_44440 E  V  V  L  L  K  *
Original_1891  GAAGTTGTATTACTTAAATAA
Optimized      GAAGTAGTTCTTTTAAAATAA
```

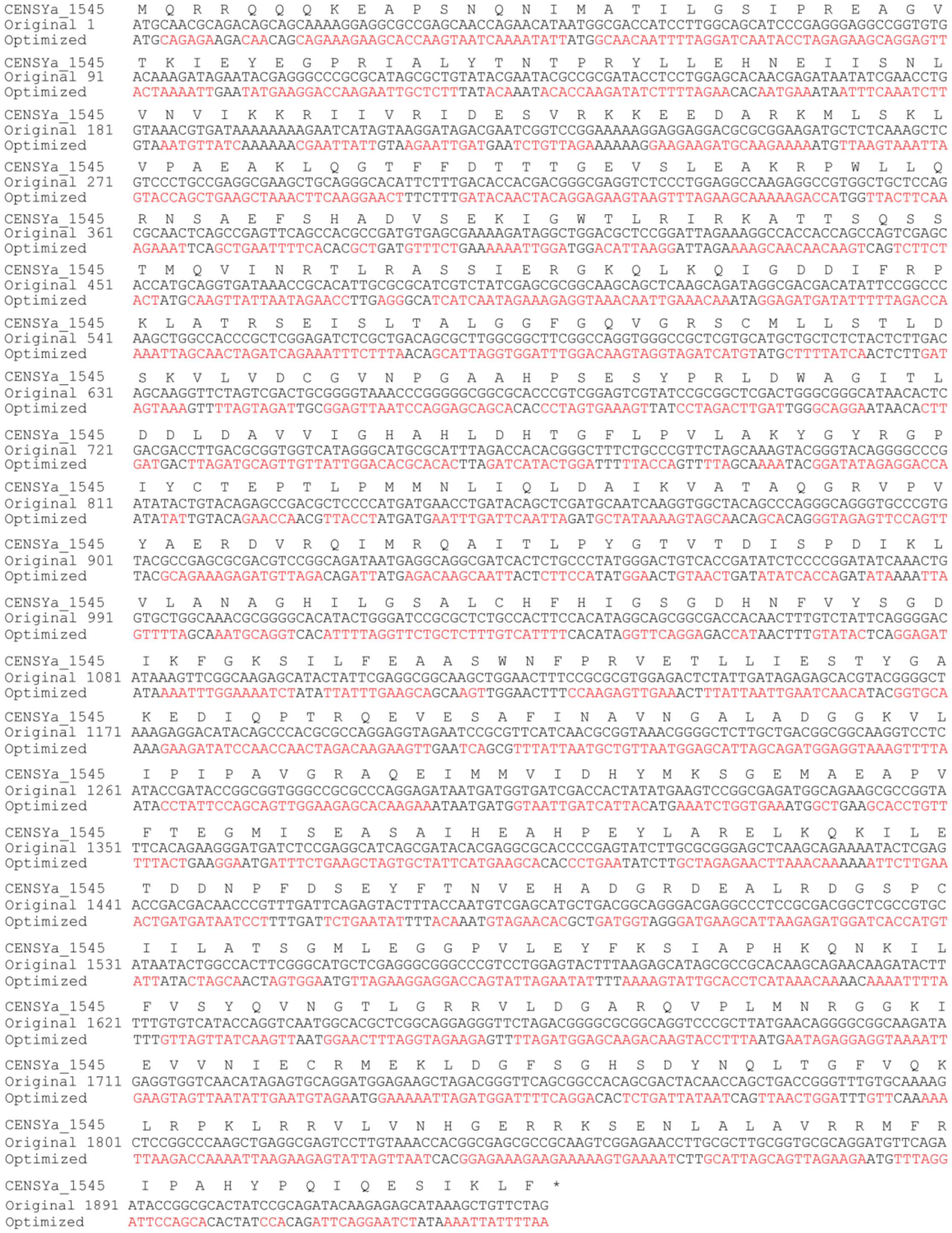

Figure S15. Codon optimization of the *aCPSF1* orthologs from *Lokiarchaeota* (*Loki-aCPSF1*: *Lokiarch_44440)* and *Thaumarcheae* (*Csy-aCPSF1*: *CENSYa_1545*). Original, the original protein coding sequences; Optimized, the optimized protein encoding sequences for compatible to the codon usage basis of *M. maripaludis*.

Supplementary Tables

Table S1. Strains and plasmids used in this study

Strains and plasmids	Characteristics and descriptions	Reference or sources
**Strains**		
E. coli DH5α	F^{-}φ80d *lac*ZΔM15 Δ(*lac*ZYA-*arg* F) U169 *end*A1 *rec*A1 *hsd*R17(r$_{K}^{-}$,m$_{K}^{+}$) *sup*E44λ- *thi* -1 *gyr*A96 *rel*A1 *pho*A	TransGen, Beijing
E. coli BL21(DE3)pLysS	F^{-} *omp*T *hsd*S(r$_{B}^{-}$m$_{B}^{-}$) *gal dcm*(DE3)pLysS Cam^{r}	TransGen, Beijing
E. coli BW25113	*lacI*^{q} *rrnB3* Δ*lacZ4787* *hsdR514* DE(*araBAD*)*567* DE(*rhaBAD*)*568 rph-1*	Prof.Tao at IMCAS
M. maripaludis S2 (*Mmp*)	Wild-type *M. maripaludis*, Pur^{S}, Neo^{S}	(4)
Mmp-tetO-aCPSF1	*hpt*::P*mcr-tetR*-T*mcr*-P*nif-tetO*-His$_{6}$-*MMP0694*, S2 with *MMP0694* inducible expression	This study
Mmp-∇*aCPSF1*	*hpt*::P*mcr-tetR*-T*mcr*-P*nif-tetO*-His$_{6}$-*MMP0694*, *MMP0694*::*pac*, Pur^{R}, strain *tetO-aCPSF1* with the indigenous *MMP0694* deletion	This study
Mmp-com(*Mmp*-C1)	*hpt*::P*mcr-tetR*-T*mcr*-P*nif-tetO*-His$_{6}$-*MMP0694*, *MMP0694*::*pac*, pMEV2-*MMP0694*, Pur^{R}, Neo^{R}, ∇*aCPSF1* with *MMP0694* complement	This study
Mmp-com(*Mmp*-C1mu)	*hpt*::P*mcr-tetR*-T*mcr*-P*nif-tetO*-His$_{6}$-*MMP0694*, *MMP0694*::*pac*, pMEV2-*MMP0694*H243/246A, Pur^{R}, Neo^{R}, ∇*aCPSF1* with *MMP0694*H243/246A complement	This study
Mmp-com(*Loki*-C1)	*hpt*::P*mcr-tetR*-T*mcr*-P*nif-tetO*-His$_{6}$-*MMP0694*, *MMP0694*::*pac*, pMEV2-*Lokiarch44440*, Pur^{R}, Neo^{R}, ∇*aCPSF1* with *Lokiarch44440* complement	This study
Mmp-com(*Csy*-C1)	*hpt*::P*mcr-tetR*-T*mcr*-P*nif-tetO*-His$_{6}$-*MMP0694*, *MMP0694*::*pac*, pMEV2-*CENSYa1545*, Pur^{R}, Neo^{R}, ∇*aCPSF1* with *CENSYa1545* complement	This study
Mmp-S2-pMEV2	pMEV2, Neo^{R}, S2 with pMEV2	This study
Mmp-aCPSF1-F	Φ(*MMP0694*-3Flag), Neo^{R}, S2 with 3Flag tags fused in the C-terminal of *MMP0694*	This study
Mmp-aRpoL-HF	Φ(MMP0261-His$_{6}$-3Flag), Neo^{R}, S2 with His$_{6}$-3Flag tags fused in the C-terminal of *MMP0261*	This study
*Mmp-*Δ*earA*	*MMP1718*::*neo*, Neo^{R}, S2 with *MMP1718* deletion	This study
Mmp-∇*aCPSF1* Δ*earA*	*hpt*::P*mcr-tetR*-T*mcr*-P*nif-tetO*-His$_{6}$-*MMP0694*, *MMP0694*::*pac*, *MMP1718*::*neo*, Pur^{R}, Neo^{R}, ∇*aCPSF1* with *MMP1718* deletion	This study
**Plasmids**		
pMD19-T	Amp^{R}	Takara, Japan
pMD19-T-*hpt*up	pMD19-T with *M. maripaludis* S2 *MMP0145* upstream fragment (*hpt*up) inserted into T-overhang end, Amp^{R}	This study
pMD19-T-P*nif-tetO*-His$_{6}$-*aCPSF1-hpt*down	pMD19-T with P*nif-tetO*-His$_{6}$-*MMP0694-hpt*down fragment inserted into T-overhang end, Amp^{R}	This study
pMD19-T-P*mcr-tetR*-T*mcr*	pMD19-T with P*mcr-tetR*-T*mcr* fragment inserted into T-overhang ends, Amp^{R}	This study
p*tetR*/*tetO*-His$_{6}$-*aCPSF1*	pMD19-T-*hpt*up with P*mcr-tetR*-T*mcr* and P*nif-tetO*-His$_{6}$-*MMP0694-hpt*down fragment inserted into 3′-ends of *hpt*up, Amp^{R}	This study
pMD19-T-*aCPSF1*up	pMD19-T with *M. maripaludis* S2 *MMP0694* upstream fragment (*aCPSF1*up) inserted into T-overhang ends, Amp^{R}	This study
pMD19-T-Δ*aCPSF1*	pMD19-T-*aCPSF1*up with the *pac* gene and *MMP0694* downstream fragment (*aCPSF1*down) inserted into 3′-ends of *aCPSF1*up, Amp^{R}	This study
pIJA03	Amp^{R}, Pur^{R}	(5)
pMEV2	Amp^{R}, Neo^{R}	(5)
pMEV2-*MMP0694*	pMEV2 with *MMP0695* promoter, 5′ UTR and ORF of *MMP0694* inserted between *Xho* I and *Bgl* II, Amp^{R}, Neo^{R}	This study
pMEV2-*MMP0694*H243/246A	pMEV2 with *MMP0695* promoter, 5′ UTR of *MMP0694* and *MMP0694*H243/246A inserted between *Xho* I and *Bgl* II, Amp^{R}, Neo^{R}	This study

pMEV2-*Lokiarch44440*	pMEV2 with *MMP0695* promoter, 5′ UTR of *MMP0694* and *Lokiarch44440* inserted between *Xho* I and *Bgl* II, Amp^R, Neo^R	This study
pMEV2-*CENSYa1545*	pMEV2 with *MMP0695* promoter, 5′ UTR of *MMP0694* and *CENSYa1545* inserted between *Xho* I and *Bgl* II, Amp^R, Neo^R	This study
pMD19-T-*aCPSF1*-3Flag	pMD19-T with *MMP0694* 3′-end fragment and 3Flag tag inserted into T-overhang ends, Amp^R	This study
pMD19-T-*aCPSF1*-3Flag-*pac*	pMD19-T-*aCPSF1*-3Flag with P*mcr*-*pac*-T*mcr* and *MMP0694* downstream fragment inserted into 3′-ends of *aCPSF1*-3Flag, Amp^R	This study
pMD19-T-*rpoL*-3Flag-His$_6$	pMD19-T with *MMP0261* 3′-end fragment, 3Flag and His$_6$ tags inserted into T-overhang ends, Amp^R	This study
pMD19-T-*rpoL*-3Flag-His$_6$-*neo*	pMD19-T-*rpoL*-3Flag-His$_6$ with P*mcr*-*neo*-T*mcr* and *MMP0261* downstream fragment inserted into 3′-ends of *rpoL*-3Flag-His$_6$, Amp^R	This study
pMD19-T-*earA*up	pMD19-T with *M. maripaludis* S2 *MMP1718* upstream (*earA*up) fragment inserted into T-overhang ends, Amp^R	This study
pMD19-T-Δ*earA*	pMD19-T-*earA*up with the *neo* gene and *MMP1718* downstream (*earA*down) fragment inserted into 3′-ends of *earA*up, Amp^R	This study
pSB1s	Str^R	provided by Prof.Tao
pSB1s-His$_6$-SUMO-*aCPSF1*	pSB1s with His$_6$-SUMO and *MMP0694* genes inserted into the expression region, Str^R	This study
pGEX-4T-1	Amp^R	
pGEX-4T-1-*rpoD*	pGEX-4T-1 with *M. maripaludis* S2 *MMP1322* inserted into *Eco*R I, Amp^R	This study
pGEX-4T-1-*rpoD/L*	pGEX-4T-1-*rpoD* with the sequence from RBS to stop codon of *M. maripaludis* S2 *MMP0261* inserted into *Xho* I, Amp^R	This study

Table S2. Primers used in this study

Primer	Sequence (5′-3′)	Purpose
*Hpt*up-F	TGTCGGGGGAGTTCAGTCC	Construction of strain ∇*aCPSF1*
*Hpt*up-R	ATGTTTTCAATGATTCTTCCAATAATT	Construction of strain ∇*aCPSF1*
P*mcr*-F	GGAAGAATCATTGAAAACATGGATGATTAATTTAAGAGA	Construction of strain ∇*aCPSF1*
P*mcr*-R	TTTATCTAATCTAGACATCATGAGAATCACTCCTATTTTTT	Construction of strain ∇*aCPSF1*
TetR-F	AAAAAATAGGAGTGATTCTCATGATGTCTAGATTAGATAAA	Construction of strain ∇*aCPSF1*
TetR-R	GGGTCGTGGGGCGGGCGTTAAGACCCACTTTCACA	Construction of strain ∇*aCPSF1*
T*mcr*-F	TGTGAAAGTGGGTCTTAACGCCCGCCCCACGACCC	Construction of strain ∇*aCPSF1*
T*mcr*-R	CTATATAAAGTTTTCGCCCCTATCAACCCAGTGAATTAAAATATAT	Construction of strain ∇*aCPSF1*
P*nif-tetO*-His$_6$-*aCPSF1*-F	TTGATAGGGGCGAAAACTTTATATAGCCCTATCAGTGATAGAGAGTTCACAACAATATATAGAGGCCTAAAAAATGCATCATCATCATCATCACTCAGCTGAAGATATATTAAACGA	Construction of strain ∇*aCPSF1*
P*nif-tetO*-His$_6$-*aCPSF1*-R	GTTTACTTTTCCGTCAACAACTTCATTATCTCAATCTTATTGAATC	Construction of strain ∇*aCPSF1*
*Hpt*down-F	ATAAGATTGAGATAATGAAGTTGTTGACGGAAAAGTAA	Construction of strain ∇*aCPSF1*
*Hpt*down-R	TCCAATGGTTCCCCCTAATC	Construction of strain ∇*aCPSF1*
19T*hpt/aC1*up-F	GCATGCAAGCTTGGCGTA	Construction of strain ∇*aCPSF1*
19T*hpt*up-R	AATTATTGGAAGAATCATTGAAAACAT	Construction of strain ∇*aCPSF1*
*aCPSF1*up-F	GATTAATATTAAGTGGTGATAAGATGAT	Construction of strain ∇*aCPSF1*
*aCPSF1*up-R	AATAGTCCCTCCTAATAATATGTCTG	Construction of strain ∇*aCPSF1*
19T*aC1*up-R	AATAGTCCCTCCTAATAATATGTCTGTTTAAATTC	Construction of strain ∇*aCPSF1*
Pac-(*aC1*up)-F	TTATTAGGAGGGACTATTATGACCGAGTACAAGCCCACG	Construction of strain ∇*aCPSF1*
Pac-(*aC1*dw)-R	AAAAGAATACTCAGGCACCGGGCTTGCG	Construction of strain ∇*aCPSF1*
*aCPSF1*dw-F	GTGCCTGAGTATTCTTTTTTTATTTTTAATTCGAATAATAGATATAC	Construction of strain ∇*aCPSF1*
*aCPSF1*dw-R	GCCAAGCTTGCATGCGTTCTGATTTCTTATGGAATTATTTATC	Construction of strain ∇*aCPSF1*
*aCPSF1Xho*I-F	CCGCTCGAGCCGGTGAGTATATAAAGCATTTATTATAAATTGATTAATATTAGGAGGGACTATTATGTCAGCTGAAGATATATTAAACG	Construction of complementary strain
*aCPSF1Bgl*II-R	GGAAGATCTTTATCTCAATCTTATTGAATCGAGA	Construction of complementary strain
pMEV2-(*Loki*C1)-F	AGATCTCATGATATCTAGATCC	Construction of complementary strain
pMEV2-(*Loki*C1)-R	CTCGAGCTCCCTGAAGAAG	Construction of complementary strain
*Loki*C1-(pMEV2)-F	TCTCTTCTTCTTCAGGGAGCTCGAGCCGGTGAGTATATAAAG	Construction of complementary strain
*Loki*C1-(pMEV2)-R	CTAGAGGATCTAGATATCATGAGATCTTTATTTTAAAAGAACTACTTCTAAGTTTTTAG	Construction of complementary strain
*Cys*C1-(pMEV2)-F	TCTCTTCTTCTTCAGGGAGCTCGAGCCGGTGAGTATATAAAG	Construction of complementary strain
*Csy*C1-(pMEV2)-R	TCCTCTAGAGGATCTAGATATCATGAGATCTTTAAAATAATTTTATAGATTCCTGAATC	Construction of complementary strain
aCPSF1-H243A-F	GTAGTAACTCACGCGGCCCTTGACCACTGTG	Site-directed mutation of *aCPSF1*
aCPSF1-H243A-R	CACAGTGGTCAAGGGCCGCGTGAGTTACTAC	Site-directed mutation of *aCPSF1*
aCPSF1-H246A-F	CGCGGCCCTTGACGCCTGTGGATTTATTC	Site-directed mutation of *aCPSF1*
aCPSF1-H246A-R	GAATAAATCCACAGGCGTCAAGGGCCGCG	Site-directed mutation of *aCPSF1*
*EarA*dw-F	AATCATGGCTTTTAATATAGATGGTTCG	Deletion of *earA*
*EarA*dw-R	TTAGTGTGTGGATGCATAACACG	Deletion of *earA*
19T*earA*dw-F	AATCATGGCTTTTAATATAGATGG	Deletion of *earA*
19T*earA*dw-R	TCTAGAGGATCCCCGGGTAC	Deletion of *earA*

*EarA*up-F	GTACCCGGGGATCCTCTAGAATGTAGATATATTTTATACGGAAGGTTC	Deletion of *earA*
*EarA*up-R	GTTCAATCATAAAAGACACCTCGAAAGTTAAAAATTAATTAAAATTAC	Deletion of *earA*
Neo-(*earA*up)-F	GGTGTCTTTTATGATTGAACAAGATGGATTG	Deletion of *earA*
Neo-(*earA*dw)-R	CTATATTAAAAGCCATGATTTCAGAAGAACTCGTCAAG	Deletion of *earA*
*aCPSF1*Flag-F	CACAGGAGACATTAAATTTGAAG	Construction of strain aCFSF1-F
*aCPSF1*Flag-R	TTATGCATAATCTGGAACATCATATGGATAAACTTTTAATTTGTCATCGTCATCTTTATAATCTTTGTCATCGTCATCTTTATAATCTTTGTCATCGTCATCTTTATAATCGATAGATCTCAATCTTATTGAATCGAG	Construction of strain aCFSF1-F
Pac-(*C1*F)-F	CCAGATTATGCATAAAATCGAAAGGAAACCTAATATGGTTTC	Construction of strain aCFSF1-F
Pac-(*C1*dw)-R	CCGATATATCTTATGCTCCTGGTTTTCTTG	Construction of strain aCFSF1-F
*aCPSF1*Flagdw-F	AGGAGCATAAGATATATCGGGGTGATACAATG	Construction of strain aCFSF1-F
*aCPSF1*Flagdw-R	CAAGCTTGCATGCCTGCAGGCGTTTTAAAGGCAATAAAATAGTATTATTG	Construction of strain aCFSF1-F
*RpoL*Flag-F	CATTGATTATTCATTTGACGCA	Construction of strain aRpoL-HF
*RpoL*Flag-R	TTAGTGGTGGTGGTGGTGGTGCTTATCGTCATCGTCCTTGTAGTCCTTATCGTCATCGTCCTTGTAGTCCTTATCGTCATCGTCCTTGTAGTCTAAATCTTCAAGGGTTTTGTTACAAAG	Construction of strain aRpoL-HF
19T*rpoL*Flag-F	CATGGTCATAGCTGTTTC	Construction of strain aRpoL-HF
19T*rpoL*Flag-R	CTGCAGGTCGACGATTTTAG	Construction of strain aRpoL-HF
Neo-(*rpoL*F)-F	CTAAAATCGTCGACCTGCAGATAAAAAACGCCCTATTCG	Construction of strain aRpoL-HF
Neo-(*rpoL*dw)-R	TATAAAATATCAATCAGAAGAACTCGTCAAG	Construction of strain aRpoL-HF
*RpoL*Flagdw-F	GTTCTTCTGATTGATATTTTATATCGGCATAATTTATTTTTTAAC	Construction of strain aRpoL-HF
*RpoL*Flagdw-R	AGGAAACAGCTATGACCATGCCCATTGTAAGTTTTGTAAATG	Construction of strain aRpoL-HF
pSB1s-F	TACAGATTAAATCAGAACGCAG	Protein expression of *Mmp*-aCPSF1
pSB1s-R	GGTTAATTCCTCCTGTTAGC	Protein expression of *Mmp*-aCPSF1
SUMO-(pSB1s)-F	GCTAACAGGAGGAATTAACCATGGGCAGCAGCCATCAT	Protein expression of *Mmp*-aCPSF1
SUMO-(*aCPSF1*)-R	CTTCAGCTGACATACCACCAATCTGTTCTCTG	Protein expression of *Mmp*-aCPSF1
aCPSF1-(SUMO)-F	CAGATTGGTGGTATGTCAGCTGAAGATATATTAAACG	Protein expression of *Mmp*-aCPSF1
aCPSF1-(pSB1s)-R	GCGTTCTGATTTAATCTGTATTATCTCAATCTTATTGAATCGAGATTCATTG	Protein expression of *Mmp*-aCPSF1
RpoD-*Eco*RI-F	GGAATTCATGAAAATGGAATTAAAAGCCCC	Protein expression of *Mmp*-aRpoD/L
RpoD-*Eco*RI-R	GGAATTCTTAATTTTCGTCACTTAATTCTTGGC	Protein expression of *Mmp*-aRpoD/L
RpoL-*Xho*I-F	CCGCTCGAGGAGGTAATTAAATATGAACTACGTTAAAATCATTG	Protein expression of *Mmp*-aRpoD/L
RpoL-*Xho*I-R	CCGCTCGAGTTATAAATCTTCAAGGGTTTTGTTACA	Protein expression of *Mmp*-aRpoD/L
P1719-NB-F	TGGGGAAACCTACGATATAGTC	Northern Blot
P1719-NB-R	GCCCCTATTTTCTTCTTCAGTAGC	Northern Blot
P1718-NB-F	TTGGGATCAGAAGACATGATATTT	Northern Blot
P1718-NB-R	ACCAGTTATCGGCAGAATCGTA	Northern Blot
P1717-NB-F	GCAGATTCTTTGAAATTATTCCTCA	Northern Blot
P1717-NB-R	TTCGACTACGATACAGTTATCTCCA	Northern Blot
PflaB2-NB-F	TTGGTACCTTGATTGTTTTTATTGC	Northern Blot
PflaB2-NB-R	TAGTGTTGTAAAGGTCTCCACCTG	Northern Blot
P1099-NB-F	TCATATCCCTGCAAAGCACT	Northern Blot
P1099-NB-R	GGAGATATGGCTTCAAGAATTGC	Northern Blot
P1100-NB-F	CAACAGTTCGTGAATCGATACTCG	Northern Blot
P1100-NB-R	TTTTACAGGAACGAACGGGA	Northern Blot
P1146-NB-F	CCAGTTCAATTTAGTTATTCTATCGA	Northern Blot
P1146-NB-R	TCCTATTTCTTTACCGTTCCC	Northern Blot
P1147-NB-F	CACGGTGAAGTAAATGACAAAAG	Northern Blot
P1147-NB-R	CTCTGTTTCCTTTTCCGGTC	Northern Blot
P1149-NB-F	CATGCTTGAAGCTATTGATGAAGG	Northern Blot
P1149-NB-R	CGATGCAAGGTACACGTC	Northern Blot

P1150-NB-F	CTTCATTTACCCCATAAGATCTTGC	Northern Blot
P1150-NB-R	CTTGGAAACGTAAGTCCTTCAGA	Northern Blot
P0155-NB-F	GTGCACTTGAAAATGGCGA	Northern Blot
P0155-NB-R	CTCTTAAGGAGTGCTTTTTTAGACC	Northern Blot
3′R-RT-P	ATTGATGGTGCCTACAG	3′RACE
1100-3′R-N1-F	TGGATAAAGAGGGCTTGGG	3′RACE
1100-3′R-N2-F	GAATGATTTGATAATGGTTGATAGACGG	3′RACE
1147-3′R-N1-F	GGAAGAAGAGCATTCCACGTAA	3′RACE
1147-3′R-N2-F	TATGTGCAGCATGCGGATTT	3′RACE
1149-3′R-N1-F	CATGCTTGAAGCTATTGATGAAGG	3′RACE
1149-3′R-N2-F	CCATCATGTTCTGCATGTATGG	3′RACE
0155-3′R-N1-F	AAAGCAGAAATGGCCGAAA	3′RACE
0155-3′R-N2-F	CAGCGTAGTTGGAAATGTTATCAC	3′RACE
0900-3′R-N1-F	GATGGAATAAAACGAATAGTTTCAG	3′RACE
0900-3′R-N2-F	GAAGCCAATCAATTGCAACG	3′RACE
0901-3′R-N1-F	CGTTCCAATTTTAGTTGCACTTAC	3′RACE
0901-3′R-N2-F	GAAGAAGATCTTGGAGAATATGAAGT	3′RACE
1241-3′R-N1-F	CACAAACAGCTACCCCGAGA	3′RACE
1241-3′R-N2-F	TGCCCAAACTGGTGAAAAATAC	3′RACE
1242-3′R-N1-F	GAACTGACGCAATCTGCAAA	3′RACE
1242-3′R-N2-F	CGATGAAAAACATAACTTTAGAAAAAATAG	3′RACE
1640-3′R-N1-F	CATCTTGTCAAACATTATTGCAGAAGA	3′RACE
1640-3′R-N2-F	GAAGGAGTAAGAGAATGTCAGATCA	3′RACE
1641-3′R-N1-F	CAGGAAGGATATACTGTAGTAATG	3′RACE
1641-3′R-N2-F	GGGGATGCTTCAAACGATATT	3′RACE
0291-3′R-N1-F	GGTGCTAAACAGATTGGGGT	3′RACE
0291-3′R-N2-F	CAGGGAAGCTGCATACACA	3′RACE
0292-3′R-N1-F	CGTTGGGATTCAGCTGTCT	3′RACE
0292-3′R-N2-F	GATACTCGGACTTATTGATACTCTTGA	3′RACE
1648-3′R-N1-F	GATATTGAATCACTATTTGGTGGA	3′RACE
1648-3′R-N2-F	GTTACGGTAATGGTTGGCC	3′RACE
1661-3′R-N1-F	GGTGAAAATGATGGAAAATAGAGA	3′RACE
1661-3′R-N2-F	GCCTTATGTTCAGAATATGGAAT	3′RACE
1719-3′R-N1-F	AAACTTACGTAGAACCCAAAACCG	3′RACE
1719-3′R-N2-F	CTACTGAAGAAGAAAATAGGGGCA	3′RACE
0295-3′R-N1-F	CCAATTTAATTGACGGATATACGGAA	3′RACE
0295-3′R-N2-F	GACATGGTCTATGGTATAACGATTAGT	3′RACE
0296-3′R-N1-F	GCACTTGTCTGGAACGAAATC	3′RACE
0296-3′R-N2-F	CGCCTGATACGATAACTGC	3′RACE
1016-3′R-N1-F	CTCTACGTTGCAGAAGGAAAAC	3′RACE
1016-3′R-N2-F	GGCGAAGAAATGGAATTAGTTGC	3′RACE
1017-3′R-N1-F	GGAGGTATGTGTAGTTTCAGTTGT	3′RACE
1017-3′R-N2-F	GAGGCAGTTTCAGAAAGCG	3′RACE
0137-3′R-N1-F	ACATTACAACAGCAGTTCCC	3′RACE
0137-3′R-N2-F	TGGGATGGGTCATTAAGCG	3′RACE
Term-3′adapter	NN-8mer_index-NNNNAGATCGGAAGAGCGTCGTGT (5′ phosphorylated, 3′ amino blocked)	Term-seq
Term-RT-P	TCTACACTCTTTCCCTACACGACGCTCTTC	Term-seq
cDNA 3′adapter	GCAGATCGGAAGAGCACACGTCTGAACTCCAGTCAC (5′ phosphorylated, 3′ amino blocked)	Term-seq
PCR-forward primer	AATGATACGGCGACCACCGAGATCTACACTCTTTCCCT ACACGACGCTCT	Term-seq
PCR-reverse primer	CAAGCAGAAGACGGCATACGAGAT-8mer_index-GTGAC TGGAGTTCAGAC	Term-seq
1100-ChIP-F	CAGCACCTTCAGAAATCG	ChIP
1100-ChIP-R	CATCAACTACAATTTTTACAGGAAC	ChIP
1149-ChIP-F	CAGAAACTTCAGAGGAAGAGA	ChIP
1149-ChIP-R	GGTTTTAATTAGATTAGAGATCTCTTG	ChIP
0901-ChIP-F	GAAGGATTAGATAAACTTGAAGAAGATCT	ChIP
0901-ChIP-R	CTTCTAAAAGGCCCATTAAACC	ChIP
0791-ChIP-F	ATGAGTTTTAACATGTATGTGCC	ChIP
0791-ChIP-R	TCTTTTGAAGTGGTTTCTTTCAA	ChIP
0136-ChIP-F	CATGTAAAAGAAGGCGACGTT	ChIP
0136-ChIP-R	CTTCAAGTGTGGCATTAATCCG	ChIP

0137-ChIP-F	GCGATATACATTACAACAGCAGTT	ChIP
0137-ChIP-R	CTCCATAAACGAGCATTGGAAAT	ChIP
0180-ChIP-F	GCAGTGAACGGAAAAGAATTAGA	ChIP
0180-ChIP-R	CTTGCAGCTCCTGCATC	ChIP
0181-ChIP-F	CATTGACATTGAAAAACGGTGG	ChIP
0181-ChIP-R	GGAAGTTCAAAATATCTGAACTTG	ChIP
0183-ChIP-F	GGACTCACCCCTATTACG	ChIP
0183-ChIP-R	GAGAATTCTTTGAGGTAGTATTCAA	ChIP
0195-ChIP-F	GGAAAGTATTGAAACGTTATCCCTTG	ChIP
0195-ChIP-R	CGTGCGACATTTTTTCATTTGT	ChIP
0210-ChIP-F	CATGGGGCTAGTAAAACAATGT	ChIP
0210-ChIP-R	GGTTGAATTTATGTATACTCCGCC	ChIP
0309-ChIP-F	CGCAATTGAAGCAGGAGC	ChIP
0309-ChIP-R	CTGCTTCTTTTACGAAAGCTATTAAAT	ChIP
0137IGR-ChIP-F	TAACAATCACAAAATACAATTTTTAAAA	ChIP
0137IGR-ChIP-R	CGAGAGTTACATAGTGTAATG	ChIP
0180IGR-ChIP-F	GTGCAGGTAAAGGTTTAAG	ChIP
0180IGR-ChIP-R	CTGTCGATAACAAGAATAGAATAT	ChIP
0183IGR-ChIP-F	CCTCGTTACAATAAACGG	ChIP
0183IGR-ChIP-R	CTATATCCTTGAATTAATAAATACGC	ChIP
1100IGR-ChIP-F	GTTCTCCAGTAATCATAAATGCAAC	ChIP
1100IGR-ChIP-R	CCGTGAAGATAGTAAAATTGACG	ChIP

Table S3. RNA-seq sequencing reads mapped to the reference genomes

Sample name	S2_1 (% of total reads)	S2_2 (% of total reads)	S2_3 (% of total reads)	aCPSF1_1 (% of total reads)	aCPSF1_2 (% of total reads)	aCPSF1_3 (% of total reads)
Total reads	19639144	14616816	16981328	17408754	16248240	16120374
Total mapped	19250607 (98.02%)	14350931 (98.18%)	16663636 (98.13%)	17073158 (98.07%)	15920263 (97.98%)	15793557 (97.97%)
Multiple mapped	471443 (2.4%)	1447395 (9.9%)	706298 (4.16%)	332895 (1.91%)	341125 (2.1%)	319736 (1.98%)
Uniquely mapped	18779164 (95.62%)	12903536 (88.28%)	15957338 (93.97%)	16740263 (96.16%)	15579138 (95.88%)	15473821 (95.99%)
Read-1	9395769 (47.84%)	6453745 (44.15%)	7984738 (47.02%)	8373575 (48.1%)	7793245 (47.96%)	7740873 (48.02%)
Read-2	9383395 (47.78%)	6449791 (44.13%)	7972600 (46.95%)	8366688 (48.06%)	7785893 (47.92%)	7732948 (47.97%)
Reads map to '+'	9390836 (47.82%)	6452743 (44.15%)	7979483 (46.99%)	8372400 (48.09%)	7791831 (47.95%)	7739061 (48.01%)
Reads map to '-'	9388328 (47.8%)	6450793 (44.13%)	7977855 (46.98%)	8367863 (48.07%)	7787307 (47.93%)	7734760 (47.98%)

Table S4. Numbers and significance statistics of the up- and down-regulated genes in each function code (as in arCOGs)

[*]Function code (as in arCOGs)[**]	C	D	E	F	G	H	I	J	K	L	
**Up**-regulated gene numbers	28	4	25	18	22	36	7	39	39	26	
**p**-Value of Up-regulated genes	1.00	1.00	1.00	1.00	0.55	1.00	1.00	0.99	0.04	0.65	
**Down**-regulated gene numbers	85	6	63	17	26	44	7	69	20	16	
**p**-Value of Down-regulated genes	<0.01	0.55	<0.01	1.00	0.16	0.65	1.00	0.06	1.00	1.00	
Total gene numbers	166	12	122	54	54	116	19	158	81	68	

Function code (as in arCOGs)	M	N	O	P	Q	R	S	T	U	V	Total
**Up**-regulated gene numbers	6	22	18	26	7	89	170	3	2	5	589
**p**-Value of Up-regulated genes	1.00	<0.01	1.00	1.00	0.46	0.55	<0.01	1.00	1.00	1.00	
**Down**-regulated gene numbers	16	6	18	33	2	62	91	4	6	8	599
**p**-Value of Down-regulated genes	0.46	1.00	1.00	0.46	1.00	1.00	1.00	1.00	0.55	0.55	
Total gene numbers	34	31	53	79	13	231	393	10	12	16	1722

[*]: The significance statistical analysis was analyzed by Fisher's exact test with Benjamini-Hochberg multiple-testing correction to calculate P-values for each function code. P-value smaller than 0.05 was thought to be statistical significance (marked by ' * ' in Fig 1d) and p-value smaller than 0.01 was thought to be extremely statistical significance (marked by ' ** ' in Fig 1d).

[**]: arCOG functional categories are described as follows according to the study: C, energy production and conversion; D, cell cycle control, cell division, chromosome partitioning; E, amino acid transport and metabolism; F, nucleotide transport and metabolism; G, carbohydrate transport and metabolism; H, coenzyme transport and metabolism; I, lipid transport and metabolism; J, translation, ribosomal structure and biogenesis; K, transcription; L, replication, recombination and repair; M, cell wall/membrane/envelope biogenesis; N, cell motility; O, posttranslational modification, protein turnover, chaperones; P, inorganic ion transport and metabolism; Q, secondary metabolites biosynthesis, transport and catabolism; R, general function prediction only; S, function unknown; T, signal transduction mechanisms; U, intracellular trafficking, secretion, and vesicular transport; V, defense mechanisms.

Table S5. Term-seq sequencing reads mapped to the reference genomes

Sample name	S2_Term_1 (% of raw reads)	S2_ Term_2 (% of raw reads)	aCPSF1_ Term_1 (% of raw reads)	aCPSF1_ Term_2 (% of raw reads)
Raw reads	19732504	19273020	22908242	26400410
Clean reads	17425060	14390894	18798980	18672222
Total mapped	11009528 (63.18%)	6556054(45.56%)	14658008 (77.97%)	13239499 (70.9%)
Multiple mapped	9266456 (53.18%)	5325929 (37.01%)	12405944 (65.99%)	11213621 (60.06%)
Uniquely mapped	1743072 (10%)	1230125 (8.55%)	2252064 (11.98%)	2025878 (10.85%)

Supplementary References

1. Miceli, R.M., DeGraaf, M.E. and Fischer, H.D. (1994) Two-stage selection of sequences from a random phage display library delineates both core residues and permitted structural range within an epitope. *J Immunol Methods*, **167**, 279-287.
2. Larkin, M.A., Blackshields, G., Brown, N.P., Chenna, R., McGettigan, P.A., McWilliam, H., Valentin, F., Wallace, I.M., Wilm, A., Lopez, R. *et al.* (2007) Clustal W and Clustal X version 2.0. *Bioinformatics*, **23**, 2947-2948.
3. Gouet, P., Courcelle, E., Stuart, D.I. and Metoz, F. (1999) ESPript: analysis of multiple sequence alignments in PostScript. *Bioinformatics*, **15**, 305-308.
4. Tumbula, D.L., Makula, R.A. and Whitman, W.B. (1994) Transformation of Methanococcus-Maripaludis and Identification of a Psti-Like Restriction System. *Fems Microbiol Lett*, **121**, 309-314.
5. Sarmiento, B.F., Leigh, J.A. and Whitrnan, W.B. (2011) Genetic Systems for Hydrogenotrophic Methanogens. *Method Enzymol*, **494**, 43-73.
